# Vasa Vasorum in Atherosclerosis and Clinical Significance

**DOI:** 10.3390/ijms160511574

**Published:** 2015-05-20

**Authors:** Junyan Xu, Xiaotong Lu, Guo-Ping Shi

**Affiliations:** 1 Second Clinical Medical College, Zhujiang Hospital and Southern Medical University, Guangzhou 510280, China; E-Mails: junyanxu_zj@126.com (J.X.); lxt0115@126.com (X.L.); 2 Department of Medicine, Brigham and Women’s Hospital and Harvard Medical School, Boston, MA 02115, USA

**Keywords:** atherosclerosis, vasa vasorum, neovascularization, mechanism, imaging modality, angiogenic therapy

## Abstract

Atherosclerosis is a chronic inflammatory disease that leads to several acute cardiovascular complications with poor prognosis. For decades, the role of the adventitial vasa vasorum (VV) in the initiation and progression of atherosclerosis has received broad attention. The presence of VV neovascularization precedes the apparent symptoms of clinical atherosclerosis. VV also mediates inflammatory cell infiltration, intimal thickening, intraplaque hemorrhage, and subsequent atherothrombosis that results in stroke or myocardial infarction. Intraplaque neovessels originating from VV can be immature and hence susceptible to leakage, and are thus regarded as the leading cause of intraplaque hemorrhage. Evidence supports VV as a new surrogate target of atherosclerosis evaluation and treatment. This review provides an overview into the relationship between VV and atherosclerosis, including the anatomy and function of VV, the stimuli of VV neovascularization, and the available underlying mechanisms that lead to poor prognosis. We also summarize translational researches on VV imaging modalities and potential therapies that target VV neovascularization or its stimuli.

## 1. Introduction

Atherosclerosis is a systemic inflammatory disease that associates with several acute cardiovascular complications triggered by atherosclerotic plaque rupture, which primarily manifests as stroke and myocardial infarction. It remains the leading cause of morbidity and mortality worldwide [1]. Considering its poor prognosis, understanding the pathophysiology of atherosclerosis and exploring potential means to discover populations at risk as well as preventing its progression remain of significant importance. For decades, postmortem evaluations have concluded that the main characteristics of rupture-prone vulnerable plaques include a thin fibrous cap, high lipid content, increased numbers of inflammatory cells, and extensive adventitial and intimal neovascularization [2,3,4,5]. Though much emphasis has been placed on intimal accumulation of lipids and inflammatory cells, recent research suggests that the adventitia vasa vasorum (VV) also plays a critical role in transforming advanced but stable lesions into vulnerable plaques at risk for rupture.

VV are defined as small blood vessels that supply or drain the walls of larger arteries and veins, delivering nutrients and oxygen as well as removing systemic “waste” products [6]. The association between VV and atherosclerotic plaque formation was first reported in 1876 by Koster [7]. Later in the 1930s, the rich vascular channels surrounding and penetrating atherosclerotic lesions, namely VV, were suspected as the source of the plaque hemorrhages for the first time [8,9]. However, the role of VV in atherosclerosis did not attract sufficient attention until half a century later when it was hypothesized that the adventitial VV of coronary arteries allowed atherosclerotic plaques to develop beyond a critical thickness by supplying oxygen and nutrients to the core of the lesions [10]. Since then, numerous studies have demonstrated the relationship between VV neovascularization and atherogenic processes. Retrospective studies on autopsies derived from humans noted that VV density was significantly increased in plaques categorized as vulnerable and prone to rupture as was those in hypercholesterolemic animal models [11,12]. In humans, it was reported that more than 80% of VV neovascularization in coronary atherosclerotic plaques had weak integrity, resulting in leakage and subsequent plaque hemorrhage [13,14]. Increased VV also associated with intimal thickening and endothelial dysfunction in animal models, and these effects could be blocked with angiogenic inhibitors [15,16]. Growing evidence supports that vascular inflammation, a crucial factor in the process of atherosclerosis, is initiated in the adventitia, and extensive inflammatory cell infiltration has been observed in the adventitial neovascular network [17]. The critical role of VV in the process of atherosclerosis has been established and identified as an independent predictor of intraplaque hemorrhage and plaque rupture. As a consequence, imaging modalities were developed to visualize VV neovascularization in the early stage of atherosclerosis. Therapies targeted to VV have emerged as a new approach for the treatment of atherosclerosis.

## 2. Structure and Function of Vasa Vasorum

### 2.1. Structure

Human VV occurs as early as the first week of gestation under an X-ray microscope, and along with the lumen blood supplement, nourishes the vessel wall [18]. VV exists mainly in the adventitial and outer medial layers of the blood vessels with more than 29 medial lamellar units or 0.5 mm lumen diameters [19]. Normal vessels in mice and intramyocardial vessels in humans do not contain VV [6].

Three different types of VV have been identified in bovine aortic walls: the VV externae (VVE), the VV internae (VVI), and the venous VV (VVV). VVE originate from major branches and the VVI originate from the main lumen of the aorta. The VVV drain the arterial wall into companion veins [20]. Direct visualizations of VV in porcine coronary arteries confirmed the coexistence of VVE, VVI, and VVV [21]. VV are often used to refer to VVE in literature because more than 96% of the newly formed microvessels in atherosclerosis sprout from VV in the adventitia and only a small part extend from the vessel lumen [22,23].

**Figure 1:**
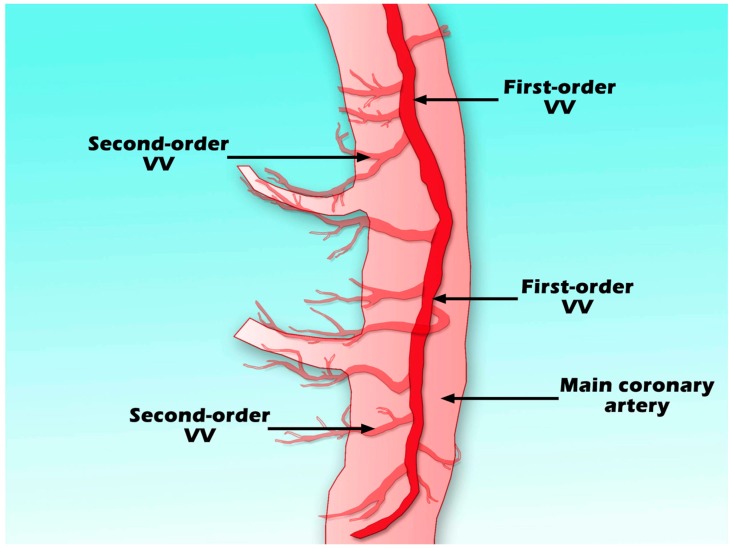
Scheme of first-order VV, second-order VV, and main coronary artery.

High-resolution micro-computed tomography (micro-CT) displayed a more precise VV structure. This technique demonstrated that VV originate from the coronary artery branch and run longitudinally along the vessel wall (first-order VV) and branch to form a circumferential plexus around the main coronary lumen (second-order VV) (Figure 1). Normal hearts had significantly greater first-order than second-order VV density (ratio 3:2), while the second-order vessel density was twofold greater than the first in hypercholesterolemic hearts [12]. Furthermore, while VV branching architecture in non-diseased porcine vasculature showed a dichotomous tree structure similar to the vasculature of systemic circulation structure, VV in diseased arteries presented many more disorganized images [21].

In atherosclerosis, previous work demonstrated that VV neovessels were immature, irregular, fragile, and prone to extravasation, particularly among those close to the atherosclerotic plaques [24,25]. Plaque neovessels showed poor coverage with mural cells and compromised structural integrity under electron microscopy, including abnormal endothelial cells (ECs), membrane blebs, intracytoplasmic vacuoles, open EC-EC junctions, and basement membrane (BM) detachment. When the mural cells are absent in either normal or atherosclerotic arteries, compromised structural integrity may cause leakage of the intraplaque microvessels [14]. Indeed, insufficient smooth muscle cell (SMC) coating in aberrant intraplaque vessels was observed in symptomatic patients when compared with asymptomatic patients [13].

### 2.2. Function

Rather than merely existing as a structural network, VV are recognized as functional end arteries that exist throughout the body [26,27]. Previous experiments demonstrate their ability to provide oxygen and nourishment to the outer third of the vascular media. Using probes to detect the diffusion of oxygen from the luminal or abluminal side of the canine femoral artery wall, the oxygen level was highest in the outer layers of the vessel wall and decreased as the probe gradually approached the lumen [28]. A more precise experiment by direct measurement of the oxygenation of the arterial wall showed the lowest level of oxygen tension of approximately 10 mmHg at about 300 μm from the lumen [29]. The varying oxygen levels between the inner and outer layers indicate that VV are the primary source of oxygen supply to the adventitia and outer media.

Along with their blood perfusion, the active exchange between VV and parent vessels meets the nutritional demands of the vessel wall and removes the “waste” products, either produced by intramural cells or introduced by diffusional transport through the luminal endothelium [6]. It is worth mentioning that VV also undertakes lipid transportation into the parent vessel wall in rabbit aorta [30], underlying a role of VV in progression of lipid core enlargement. It was also speculated that VV can regulate their own tone and vascular perfusion because the proximal VV form a regularly layered vascular structure of ECs, vascular smooth muscle cells (VSMCs), and surrounding connective tissue [31]. VV dissected from porcine and canine aorta are able to contract and dilate when exposed to endothelin-1 (ET-1) and several other vasodilators [32,33]. VV also participates in restoring the injured vessels [34] and thus are involved in several pathological conditions, including atherosclerosis, abdominal aortic aneurysm (AAA), and pulmonary artery hypertension. In fact, VV were described as “rich vascular channels surrounding and penetrating sclerotic lesions” via injecting India ink into the coronary artery wall, suggesting a role of VV in promoting atherogenesis [35]. Prior evidence suggests that VV neovascularization is a simple reaction that helps meet the demands of the intima and inner layers of the media in response to decreased oxygen concentration and malnourishment. This process gravitates towards preserving the integrity of atherosclerotic plaques. In advanced stages of atherosclerosis, however, the newly formed microvessels become important channels for various inflammatory cells and enable cellular migration to the intima, therefore becoming detrimental to the plaque integrity [36]. Plaques with a high density of neovessels are at a higher risk of hemorrhage, expansion, atherothrombosis, and rupture. As the channels conveying erythrocytes, lipids and inflammatory cells, VV neovessels seem to be multifaceted in atherosclerosis.

## 3. Stimuli of Vasa Vasorum Neovascularization

VV neovascularization is essential to atherogenesis. Metal cannulae-tied transparent visualization of human heart coronary arteries established an association of VV in atherosclerosis progression and associated sequelae [10]. High-fat diet-fed monkeys with atherosclerosis experienced blood flow from VV to the intima-media that was 10 times greater than monkeys on a normal diet. Reduced blood flow in VV directly associated with atherosclerosic lesion regression in monkeys [37]. Since these earlier pioneering studies, accumulating evidence has proven the role of VV neovascularization in both the initiation and progression of atherosclerosis, although many observations in VV neovascularization initiation and stimulation still remain incompletely understood. The extent and distribution of the ectopic neovascularization within the arterial wall depend on a number of physiological and pathological factors.

### 3.1. Hypoxia

Hypoxia and its role in the progression of VV neovascularization have been broadly studied in both cardiovascular and pulmonary arteries. Atherosclerosis, AAA, pulmonary artery hypertension, and many other systemic/pulmonary vascular diseases closely relate to hypoxia and its secondary complications [38,39]. Many factors contribute to the generation of a hypoxia environment.

Intimal thickening is an immediate element that causes hypoxia and the most prominent feature of atherosclerosis throughout the lesion initiation, progression, and ultimate rupture [40,41]. Insufficient arterial oxygen supply due to intimal thickening may shorten oxygen and nutrient diffusion distance between the deep layer of the intima and the luminal surface, resulting in regional hypoxia, ischemic injury of the inner arterial wall, and the eventual induction of VV neovascularization [42]. Increased intimal thickening and plaque growth will enhance the area and degree of hypoxia. Meanwhile, decreased oxygen supply and nourishment of the vessel wall can also originate from the changes VV undergo. As functional end arteries, VV are especially vulnerable to hypoxia [43]. An important consequence of the architecture of VV is that the blood supply cannot reach far enough from the adventitia into the media due to the pressure within the arterial wall, according to Lamé’s Law [44]. Several risk factors affect VV blood flow and lesion hypoxia. Aging, compression, or hypertension inevitably increase the arterial vessel tensile force and interfere with VV blood circulation to the inner layers, leading to low oxygen concentration in VV capillaries [6]. Smoking or nicotine inhalation is another known risk factor that contracts the peripheral arteries, thereby reducing the peripheral blood flow as well as blood flow in VV. All these risk factors are common among patients with atherosclerosis. Therefore, poor blood supply from VV induces a hypoxia environment in the intima and part of the media. An increase of lesion oxygen consumption is another risk factor of hypoxia. An active metabolic process within the cholesterol-containing macrophages and foam cells in the lesions contribute to hypoxia by increasing oxygen consumption. Bjönrnheden *et al.* [45] demonstrated that oxygen consumption was increased in foam cells isolated from the aortic intima-media in atherosclerotic rabbits. Further experiments confirmed that hypoxia correlated with the presence of macrophages and angiogenesis in advanced human carotid plaques, suggesting that hypoxia depended more on the high metabolic demand of lesion inflammatory cells than the vessel wall thickness [46]. Therefore, impaired oxygen diffusion capacity due to intimal thickness, reduced VV blood circulation, and increased oxygen consumption in atherosclerosis together generate an oxygen-insufficient microenvironment.

As a compensatory reaction to the hypoxia, VV tend to sprout across the arterial wall toward the vessel lumen to support the inner layers, called lumenward, according to Zemplenyi *et al.* [42]. In balloon-injured arteries, in which the oxygen supply in the arterial wall is impaired, newly formed VV may compensate the shortage of oxygen supply [46,47]. Spatial VV contents were increased from the dense microvessel network in the adventitia and extended to plaques in the presence of atherosclerosis [48,49]. There was an inverse correlation between low VV contents and decreased oxygenation (*i.e.*, increased expression of hypoxia-inducible factor (HIF)-1α) and increased oxidative stress (*i.e.*, increased superoxide production) within the coronary vessels in atherosclerotic pigs [50]. Additionally, prior studies suggest the signaling pathway of hypoxia-induced angiogenesis is mediated partially via regulating the production of HIF and its downstream factors. HIF is a key regulator of atherosclerosis. It affects multiple pathological events in atherogenesis, including foam cell formation, cellular proliferation, plaque ulceration, lesion hemorrhage, and rupture [51]. HIF-1 is considered the most interrelated factor of the angiogenic process in atherosclerosis. It is a basic helix-loop-helix heterodimer containing HIF-1α and HIF-1β that activates the transcription of hypoxia-inducible genes, such as erythropoietin, vascular endothelial growth factor (VEGF), E26 transformation-specific-1 (Ets-1), heme oxygenase-1 (HO1), inducible nitric oxide synthase (iNOS), and the glycolytic enzyme aldolase A [52,53]. Among them, VEGF and Ets-1 are important regulators of hypoxia-induced angiogenesis via regulating the biology of ECs.

The VEGF family has potent mitogenic and promigratory actions specific for ECs, leading to the conversion to angiogenic phenotypes, which link tightly to neovessel development in both physiological and pathophysiological conditions [54]. VEGF-A is the major subtype of the VEGF family and plays a pivotal role in the induction of neovessels through binding and primarily activating the VEGF receptor type-2 (VEGFR-2, also called KDR or Flk-1) [55]. VEGF acts as a hypoxia-inducible factor [56]. In hypertensive rat aorta, the expressions of VEGF and HIF-1α change concurrently and associate with arterial VV formation. Such a relationship was also reported in atherosclerotic lesions [57]. VEGFR-3 is a receptor for VEGF-C and VEGF-D [58,59]. Although the current consensus is that VEGFR-3 is expressed restrictedly in lymphatic vessels and can induce lymphatic EC proliferation [60,61], an underlying relationship between VEGFR-3 and VV has also been proposed. Immunostaining demonstrated the existence of VEGFR-3 in the VV of the adult aorta and other fenestrated blood vessels in human tissues, such as bone marrow, splenic and hepatic sinusoids, kidney glomeruli, and endocrine glands [62]. In human aortas, VEGF-D is constitutively expressed in normal, fatty streak, and atherosclerotic lesions, as confirmed by both immunostainings and *in situ* hybridization. In atherosclerotic lesions, VEGFR-2 is expressed in SMCs and ECs from the intima, media, and adventitia, whereas VEGFR-3 mainly exists in ECs from the adventitia, which is rich in neovascularization. The VEGF-D/VEGFR-2 cascade was probably the prominent trigger in promoting atherosclerotic plaque neovascularization [63]. From a different study, immunostaining located VEGFR-2 to the luminal endothelium in human atherosclerotic lesions, whereas VEGFR-3 was expressed in SMCs from the media and adventitia, but not in the luminal endothelium [64]. Two studies showed different expression patterns of VEGFR-3 in human atherosclerosis lesions, suggesting that VEGFR-3 has functions other than neovascularization. In a mouse atherosclerosis model, transgenic expression of soluble VEGFR-3 or its mutant did not affect atherosclerotic lesion adventitial VV density, but nearly completely blocked the lymphoid vessel growth, leading to increased plasma cholesterol and triglyceride levels and enhanced atherosclerosis [65]. These studies suggest that VEGFR-3 contributes to atherosclerosis by interacting with VV in addition to lymphoid vessels.

**Figure 2:**
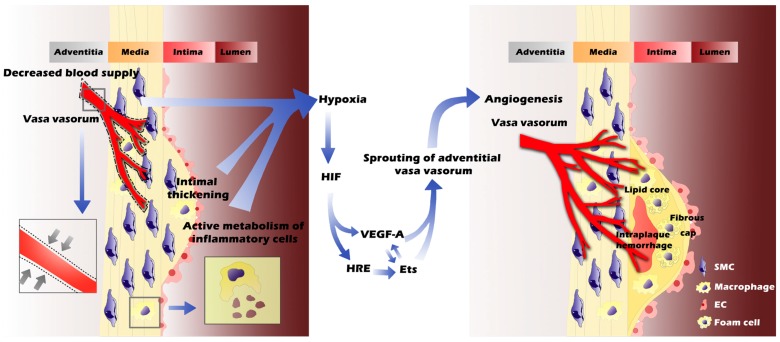
Intimal thickening, decreased blood supply (due to high pressure in parent vessel and the stimulated constriction of VV, represented by grey arrows in the left closed box) and active metabolism of inflammatory cells together contribute to hypoxia in atherosclerotic vessels (**left** panel). The oxygen-insufficient microenvironment in inner layers of vessel wall further induces angiogenesis through activating HIF, VEGF-A and Ets signaling pathways. As a result, the formation of intraplaque neovessels originating from VV leads to the progression of atherosclerotic plaques, including intraplaque hemorrhage, lipid core enlargement, inflammatory cell infiltration, and ultimate rupture (**right** panel).

Evidence shows that the Ets transcription factor regulates the expression of matrix metalloproteinase (*MMP*) genes, which are related to the essential steps in angiogenesis and tumor invasion (extracellular matrix degradation and vascular EC migration) [66]. Hypoxia induces Ets-1 expression via the activity of HIF-1 [53], likely via the hypoxia-responsive element (HRE) located at the Ets promoter. In addition to inducing protease expression, Ets-1 also influences angiogenesis by enhancing the transcription of hepatocyte growth factor (HGF) and VEGF, and forming an auto-loop of their upregulation [67]. It is speculated that the role of Ets-1 in atherosclerosis may have the same activities. Indeed, HIF-1, VEGF, and Ets-1 were all expressed in 29 human carotid plaques obtained from carotid endarterectomy [68]. Hypoxia-induced HIF-1a/VEGF/Ets-1 cascade was suggested as important for angiogenesis in human atherosclerosis. Nevertheless, the connections among these hypoxia-induced angiogenesic pathways still remain unclear. For example, in contrast to the studies discussed above, acidic fibroblast growth factor (FGF), basic fibroblast growth factor (bFGF), VEGF, and epidermal growth factor all induce the expression of Ets-1 mRNA in ECs [69,70], suggesting that multiple mechanisms can contribute to hypoxia-induced angiogenesis. Therefore, whether VEGF and Ets play an independent role in the process of VV neovascularization, or act synergistically with other growth factors, merits further investigation (Figure 2).

### 3.2. Inflammation

Systemic atherosclerosis, parenchymal inflammation and VV neovascularization are inseparably linked. There are two main hypotheses for the initiation of vascular inflammation. The traditional concept of vascular inflammation includes “inside-out” responses centered on the monocyte adhesion and lipid oxidation hypotheses. However, growing evidence supports a new paradigm of an “outside-in” hypothesis, in which vascular inflammation is initiated in the adventitia and progresses inward toward the intima [17]. Although the initiation of vascular inflammation is still up for debate, VV neovascularization is no doubt triggered and perpetuated by inflammatory reactions within the vascular wall [71]. In fact, the role of inflammation in neovessel formation was proposed decades ago [72]. VV neovascularization occurs most prevalently at the sites of the intima that contain chronic inflammatory cell infiltration, especially the macrophages and lymphocytes. Furthermore, inflammatory cytokines, growth factors, and angiogenic stimuli, which are released by activated inflammatory cells (e.g., macrophages), can enhance not only the inflammation itself but also the development of VV neovascularization [73]. Some studies suggest that vascular inflammation in atherosclerotic lesions closely associates with cell metabolism-created hypoxia, microvascularization, hemorrhage formation, and plaque rupture [74]. However, the exact mechanisms of the inflammation-induced angiogenesis remain unknown.

### 3.3. Lipids

The role of lipids in atherosclerosis is an old topic. Several types of lipid complex or lipid-containing substances have been reported in atheromatous lesions, including modified oxidized-LDL, 7-ketocholesterol (7KCh), cholesterol (such as low-density lipoprotein LDL, high-density lipoprotein HDL, and triglyceride), soluble phospholipids, and eicosanoids [75,76]. These lipid complexes or lipid-containing products can be present in the circulation, released from dead foam cells, or exist on the blood cell membrane. High levels of circulating LDL remain a profound risk factor in predicting cardiovascular events. Circulating LDL is an important source of atherosclerotic plaque lipid content and contributes to atherosclerosis progression, such as lipid deposition/foam cell formation and associated inflammatory process [77]. As the disease progresses, lipid-laden foam cells undergo apoptosis and release free cholesterols, leading to the formation of necrotic cores full of lipids [78]. Studies of red blood cell membrane components present another conceivable origin of lipids in atherosclerosis [79]. Taken together, atherosclerotic plaque is recognized as a reservoir of lipid complexes.

VV neovascularization begins at the radial projection at the site of lipid retention [80]. It provides atherosclerotic lesions with blood as well as lipids [81]. Hypoxia may not be the only factor responsible for medial VSMC proliferation or activation due to the restricted hypoxia in intimal lesions [40]. Therefore, experts hypothesize that VV neovascularization may be lipid-dependent. Indeed, intima-borne lipid promotes angiogenesis by activating VSMC PPAR-γ receptors [76]. Conditioned medium from early atheromatous lesions was enriched in oxidized lipid 15-Deoxy-δ-12, 14-prostaglandin J2, and their derivatives. These naturally occurring compounds activate the PPAR-γ pathway in subjacent medial VSMCs [82]. In pace with PPAR-γ activation, the expression of VEGF-A was upregulated in VSMCs, leading to neovascularization. Cholesterol efflux also regulates angiogenesis via the modulation of lipid rafts and VEGFR-2 signaling in ECs. The decline of the lipid rafts results in lower VEGFR-2 contents on the cell membrane, which leads to the down-regulation of VEGFR signaling and culminates in the inhibition of VEGF-stimulated angiogenesis [83]. This might be an entirely new hypothesis to interpret the modulation of the lipid-mediated angiogenic process. These results were all based on the model of dyslipidemia zebrafish, which might be a better model for the study and evaluation of the early development of atherosclerosis. Besides the aforementioned VEGF-dependent mechanism of angiogenesis, the VEGF-independent pathway also contributes to the angiogenesis in vascular disease. Polyunsaturated fatty acid (PUFA), which is prone to oxidation, is capable of activating the Toll-like receptor 2 (TLR2)/MyD88 pathway after oxidation. This leads to the activation of Rac1 to promote NF-κB signaling, causing cell migration and neovascularization [84,85]. Nevertheless, more mechanisms may come to explain VV neovascularization and each mechanism may not be independent but instead may regulate each other.

Growing evidence indicates that the perivascular adipose tissue (PVAT) associates with the inflammatory process in atherosclerosis. PVAT interacts directly with the outer adventitia without fascia or elastic lamina and is capable of conveying signaling molecules (adipokines and cytokines) to the adjacent blood vessels [86]. VV, which penetrate the PVAT, are highly prone to change. Conditioned medium from differentiated murine 3T3-L1 adipocytes concentration-dependently stimulates human saphenous vein and aortic SMC proliferation. There was an about 206% ± 21% increase of SMC proliferation in the vein and 145% ± 9% SMC proliferation increase in the aorta at the highest concentration (100 μL/mL) used, while such an effect was not observed in conditioned medium from premature or undifferentiated adipocytes [87]. Experts concur that adipokines such as visfatin and leptin are the stimuli in SMC proliferation and migration [88]. Compared with conditioned medium from differentiated human subcutaneous and perirenal adipocytes, conditioned medium from differentiated human perivascular adipocytes showed a much stronger ability to induce angiogenesis (elongation and branching) when applied to human coronary artery ECs, consistent with the elevated (two-fold) expression of VEGF in perivascular adipocytes [89]. In advanced stages of atherosclerosis, neovessels were suspected as the conduit for transporting pro/anti-inflammatory mediators into the vascular wall from PVAT. However, since adipocytes are heterogenous in different tissues and even PVAT are biologically and functionally diverse surrounding different blood vessels [90], a detailed relationship between different PVAT locations and properties remain to be tested.

## 4. Factors Leading to Immature and Fragile Vasa Vasorum

Although VV neovascularization is generally recognized as a compensatory reaction to meet the oxygen and nutritional demand of the inner layer of the vascular wall, intraplaque neovascularization meanwhile gives rise to ensuring plaque destabilization, intraplaque hemorrhage (IPH), atherothrombosis and even ultimate plaque rupture. As mentioned, intraplaque VV are immature, irregular, fragile, and prone to extravasation due to the compromised structural integrity. Different hypotheses have been proposed to explain this immaturity and leakage in varying stages and timescales in atherosclerosis.

### 4.1. Imbalance of Angiogenic Factors in Proteolytic Environment

Atherosclerotic plaque contains a wide spectrum of proteolytic proteases, including metalloproteinases (*i.e.*, MMPs), serine proteases (e.g., elastase, coagulation factors, plasmin, tissue-type plasminogen activator and urokinase-type plasminogen activator), and cysteine proteases (e.g., cathepsins) [91]. Previous studies have demonstrated their tight correlation to the pathophysiology of atherosclerosis, in particular, concerning the plaque destabilization. The MMP family is involved in intraplaque angiogenesis and plaque instability. Increased expression of MMP-1, -2, -3, and -9 was detected in atherosclerotic plaques [92]. Urokinase-type plasminogen activator receptor (UPAR) expression was 1.4-fold higher in macrophages and 1.5-fold higher in carotid endarterectomies from Caucasian patients with symptomatic carotid stenosis, compared to the control group [93]. In unstable plaques, increased legumain was detected and converted cathepsin L to its mature 25 kDa form, leading to the intraplaque angiogenesis, macrophages apoptosis, and necrotic core formation [94,95].

In human carotid endarterectomy samples, the levels of placental growth factor (PlGF), VEGF, and angiopoietin-1 (Ang-1) were significantly decreased in culprit plaques/hemorrhagic when compared with culprit plaques/non-hemorrhagic, but both were higher than those from the normal control group. Soluble Tie-2 (receptor of Ang-1 and Ang-2) levels were also increased in the hemorrhagic lesions, although Ang-2 levels were similar between hemorrhagic and non-hemorrhagic lesions [96]. These results suggested an angiogenic/anti-angiogenic imbalance in hemorrhagic plaques. Since the normal formation of neovessels requires a precise regulation to maintain the balance between angiogenic and anti-angiogenic factors, the disturbed balance in hemorrhagic plaques may impede the maturity and structural integrity of intraplaque neovessels.

Previous studies revealed a significant increase in plasmin and leucocyte elastase activities in hemorrhagic plaques [97]. These proteases may degrade these angiogenic factors in hemorrhagic plaques, resulting in angiogenic/anti-angiogenic imbalance and potential neovessel immaturity. VEGF promotes the initiation of immature vessels by vasculogenesis or angiogenic sprouting. Paracrine Ang-1 stabilizes the interactions between ECs and their surrounding support cells (SMCs and pericytes) and extracellular matrix (ECM) via binding to the Tie 2 receptor on the EC surface. Autocrine Ang-2, considered as an antagonist for Tie 2, has adverse effects, leading to vascular regression or angiogenic sensitivity (more plastic and destabilized state) [98]. Alteration of Ang-1/Ang-2 influences the normal process of neovessel formation. Although a shift of Ang-1/Ang-2 ratio was observed in several pathological processes, such as brain arteriovenous malformations [99] and tumor microvessel development [100], its role in atherosclerosis remains largely unknown. The imbalance between Ang-1 and Ang-2 in atherosclerosis may be a major deterrent to neovessel maturation. Decreased activity of Ang-1 and the ratio between Ang-1 and Ang-2 levels biased towards Ang-2 were observed in atherosclerotic plaques along with high microvessel content [101]. This is consistent with the observation that plaques with high microvessel density are at a high risk of intraplaque hemorrhage [102]. In addition, the Ang-1/Ang-2 ratio in favor of Ang-2 was also observed in hemorrhagic plaques, indicating an underlying role of angiopoietin/Tie system in microvessel immaturity [96].

Therefore, angiogenesis may depend on a precise balance of positive and negative regulations. The angiogenic and anti-angiogenic factors act in coordination to form well-structured and functional vessels. A disorder of homeostasis in VV neovascularization influences the proliferation and migration of ECs and their surrounding support cells, thereby leading to compromised structural integrity and aberrant neovessel formation. However, angiogenic factors can be regulated at the levels of both expression and proteolytic degradation. It remains unknown how these angiogenic factors are regulated in VV neovascularization during atherogenesis, a possible focus of future studies.

### 4.2. Further Exacerbation of Neovessel Damage: Iron, Cholesterol Crystal and Proteases

Angiogenic/anti-angiogenic imbalance inherently leads to the extravasation of vessel wall. Iron, cholesterol crystal, and protease activity may cause further exacerbation of neovessel damage, which also facilitates the permeability of intraplaque microvessels. Iron is abundant in the human body, especially in erythrocytes. Differing from normal erythrocytes, erythrocytes in atherosclerotic plaques are prone to undergo rapid lysis to release a large quantity of hemoglobin (Hb) [103]. Extracellular Hb remains susceptible to morphing into ferrihemoglobin via oxidation, and releases heme that contains abundant redox active iron. Redox active iron plays a detrimental role in oxidation reactions *in vivo*, including lipid oxidation. Oxidized LDL from atherosclerosis is cytotoxic to ECs [104,105]. High expression of ferritin and heme oxygenase-1 that control the redox active iron and degraded heme demonstrate a protective compensatory reaction from iron-derived vascular injury [106,107]. Diaspirin cross-linked Hb (DBBF-Hb) and polyethylene glycol (PEG)-conjugated Hb, proposed as blood substitutes, proved to increase arterial microvascular permeability [108]. Therefore, erythrocyte-derived iron may induce vascular damages, establishing a role for heme iron-dependent oxidation in damaging intraplaque immature microvessels. Apart from erythrocyte-derived iron, several other mechanisms, which are not mutually exclusive, have been proposed as underlying causes of vessel injury within plaques, including perforation of microvessels by cholesterol crystals [109] and direct damage induced by protease activity [110].

## 5. Vasa Vasorum Imaging in Atherosclerosis Plaques

Atherothrombosis resulted from plaque rupture is the direct cause of several acute cardiovascular complications (e.g., stroke, myocardial infarction). Therefore, practical imaging systems for early detection of unstable or even asymptomatic atherosclerotic plaques are urgently needed. Angiographic studies showed that non-obstructive plaques caused approximately 75% of cases with acute coronary occlusion [111]. Therefore, traditional focus on the stenosis of atherosclerotic plaque may be not sufficient to predict vulnerable plaques, thereby forcing us to explore more detailed features of possible culprit plaques, such as the fibrous cap, a lipid-rich necrotic core, signs of inflammation, and VV neovascularization. Visualization of arterial VV and intraplaque neovessels has recently emerged as a new surrogate marker for the early detection of atherosclerotic lesions. Coronary VV neovascularization occurs within the first week of experimental hypercholesterolemia, prior to the development of endothelial dysfunction of the host vessel, suggesting the significance of VV visualization in the identification of atherosclerotic vascular disease in the early stage [16]. Thus, a safe, non-invasive, and affordable imaging technique for the detection of VV has important clinical significance.

### 5.1. Anatomic Imaging of Vasa Vasorum in Atherosclerosis

Differences in VV structure between normal and atherosclerotic arteries were first detected by autopsy cinematography. The 3D anatomy and tree-like branching architecture of VV are shown in Figure 1. Efforts have been focused on identifying not only diseased and non-diseased arteries, but also stable and unstable plaques among risky populations through anatomic imaging.

Growing evidence confirming the role of VV neovascularization in atherosclerosis is provided first by micro-CT. With micro-CT, increased VV contents in the proximal left anterior descending coronary artery were detected in hypercholesterolemic pigs [48]. VV density was found about two times higher in nonstenotic and noncalcified stenotic plaques when compared with normal and calcified segments [11]. With the limitation of the subject imaging volume, micro-CT is mostly used to scan specimens from autopsy or small animals such as mice. Thus, micro-CT usually applies to retrospective studies or animal experiments, and is not available for humans.

High-speed whole body scanning capability of multi-slice CT demonstrated its capacity of accurate visualization of atherosclerotic plaques in the coronary arteries [112]. Several attempts have been made to improve the resolution of CT in VV visualization. For example, iodinated nanoparticles were used as contrast agents in CT. CT imaging using this compound increased the X-ray absorption at the targeted sites, hence improving the quality of CT images. CT angiography (CTA) is another technique with high spatial and temporal resolution, allowing detailed anatomical delineation of atherosclerotic arteries. CTA scanning of patients with moderate (50%–70%) stenosis of the internal carotid artery showed that plaques derived from patients with neurological symptoms had a higher proportion of VV enhancement than of that in total patients (34% *vs.* 24.1%), which indicated that atherosclerotic patients with enhancing VV were more likely to be symptomatic [113]. This result indicates that CTA imaging of VV may aid in the identification of patients at an increased risk for ischemic stroke within populations with the same degree of stenosis. However, because of the risks associated with radiation exposure, current American Heart Association and American College of Cardiology guidelines do not recommend CTA as a general screening tool in low-risk, asymptomatic patients [114].

Intravascular ultrasound (IVUS) is broadly used to provide high-resolution tomographic images of the lumen and acquire precise measurements of atherosclerotic plaques *in vivo*. However, IVUS imaging systems, which are developed to examine blood flow within the lumen of large arteries, are not designed initially to detect VV morphology [115]. Recently, a porcine experiment demonstrated that the change of IVUS-based vessel wall flow assessment signals paralleled VV density detected by micro-CT, indicating the potential of IVUS estimation of blood flow to quantify VV density [116]. Contrast-enhanced IVUS with contrast enhancement agents is another prominent method used in VV visualization. Contrast agents can increase IVUS echogenicity enhancement in the adventitia of coronary arteries, which is consistent with the enhancement of VV [117]. O’Malley *et al.* [118] presented analyses of human coronary arteries *in vivo*, and demonstrated the feasibility of contrast-enhanced IVUS imaging of VV density and perfusion in atherosclerotic plaques. Further, IVUS with contrast microbubbles tracing neovascularization in non-culprit coronary atherosclerotic plaques demonstrated a significant mean enhancement after intracoronary injection of microbubbles (from 7.1% ± 2.2% to 7.6% ± 2.5%) in the adventitia, which represented the high density of VV in patients with acute coronary syndrome [119]. Other modified techniques, such as contrast-harmonic IVUS and subharmonic contrast IVUS, can visualize contrast agents in adventitial VV [120,121]. Compared with harmonic contrast IVUS, subharmonic (SH20) imaging was even superior to harmonic (H40) imaging in terms of contrast-to-noise and contrast-to-tissue ratio improvement [122]. However, quantitative comparison of harmonic and subharmonic imaging has not been available. Although contrast-enhanced IVUS can provide clear and direct insight into VV in the adventitia, experiments that quantify the neovessels are not available, leading to the limitation of IVUS application in clinical practice. A more accurate index is needed to visualize adventitial VV.

Contrast-enhanced ultrasonography (CEUS), another modality for vascular imaging together with ultrasonographic contrast microbubbles, has developed during the last decade. In a preliminary feasibility study, CEUS enabled the visualization of the adventitial network of VV in human carotid arteries [123]. The enhanced signal was five times higher on average after stimulating atherosclerosis [124,125]. These enhancements correlated with the histological density of intraplaque neovessels. Visualization of VV density by CEUS in an atherosclerotic population also revealed a positive relationship between the abundance of VV and plaque echolucency, a well-accepted marker of high risk lesions [126]. The capability of CEUS in visualizing adventitial VV and intraplaque neovascularization makes it attractive for plaque risk stratification and assessment of anti-atherosclerotic therapy efficacy. Significant linear correlations between CEUS peak video-intensity and histologic VV counting, as well as the cross-sectional area of neovessels were recently reported [127,128]. Video intensity has become a quantitative parameter of CEUS to detect VV and assess the effects of anti-atherosclerotic therapy. Normalized maximal-video intensity enhancement (MVE) in CEUS, which represented the density of VV, demonstrated a positive relationship with plaque volume. A much lower MVE enhancement was observed after four weeks in atorvastatin-treated rabbits (from 0.18 ± 0.08 to 0.16 ± 0.07, *p* = 0.11) than in untreated rabbits (from 0.18 ± 0.08 to 0.25 ± 0.08, *p* = 0.001) [129]. In humans, quantitative CEUS was applied to investigate coronary artery disease patients undergoing lipid-lowering therapy with statins, which parelleled the LDL reduction [130]. All these results inspire the development of a standard diagnostic index for CEUS imaging in quantifying VV density, with which we could identify the populations with unstable plaques and plan the medication. To achieve this goal, large multicenter clinical trials on quantitative CEUS scanning of VV in normal and diseased populations are needed. However, a controversial study indicated that the enhancement of CEUS in carotid atherosclerotic plaques might not always reflect the presence of VV, as verified by the immunochemistry results [131]. Thus, more studies are needed to improve imaging quality and certain clinical standards of CEUS in VV visualization and vulnerable plaques prediction.

Optical coherence tomography (OCT) is an intravascular imaging modality using near-infrared light to generate cross-sectional intravascular images [132]. With its high resolution (10–20 µm), which is 10 times higher than that of IVUS and comparable to that of micro-CT, OCT is widely used for the assessment of coronary atherosclerotic plaques [133]. Under OCT, VV are visualized as a no-signal microchannel within the plaque or the adventitia [134]. The greatest challenge for VV detection under intravascular modalities is the extensive motion artifacts inherently associated with arterial pulsations in addition to other physiological movements. These limitations have been minimized in a recent intensity kurtosis OCT technique, which was developed to visualize VV from carotid arteries *in vivo* [135]. Both the blood flow into VV and dynamic motions of the arterial wall were clearly displayed using this OCT technique. In addition to the earlier time-domain OCT, optical frequency domain imaging (OFDI) was developed as a new-generation OCT that was capable of obtaining A-lines at much higher imaging speeds, facilitating rapid, 3D-pullback imaging during the administration of a non-occlusive flush of an optically transparent media such as Lactated Ringer’s or radiocontrast [136]. The laboratory results showed that adventitial VV on OFDI *ex vivo* were clearly displayed and appeared to communicate with the coronary adventitia and media. The results were compatible with histological findings and showed much better resolution when compared with other generations of OCT [137]. More recently, quantification of VV with the 3D OCT method has been applied in both animal and clinical studies. Animal studies conducted on swine suggest significant correlations between the microchannel volume (MCV) count by OCT and the amount of VV by micro-CT [138], which was consistent with the human study [137]. A positive correlation between MCV and plaque volume was also detected from this study [138]. In 2012, international guidelines were formed by the International Working Group for Intravascular OCT Standardization and Validation. OCT imaging modality was recommended as a standard reference in clinical practice [136].

### 5.2. Molecular Imaging of Vasa Vasorum in Atherosclerosis

The recent recognition that plaque biological features influence the prognosis of atherosclerosis has led to the transition from the sole anatomical assessment to combined anatomic and functional imaging modalities, enabling the application of molecular imaging in VV detection. Molecular imaging, which originates from cancer imaging, is defined as *in vivo* characterization and the measurement of biological processes at the cellular, molecular, whole organ, and whole body levels [139]. Radionuclide tracers or targeted agents with specific binding capacity to molecular targets were used as markers of biological functions in molecular imaging.

Nuclear positron emission tomography (PET) and single-photon emission computed tomography (SPECT) were the first two methods used for molecular imaging. These techniques mostly detected the hypoxia and inflammation levels of atherosclerotic plaques through fluorodeoxyglucose (FDG) uptake or other targets [140,141]. As for the angiogenesis scanning, PET/SPECT with ^64^Cu-labeled VEGF_121_, ^111^In-labeled αvβ_3_-targeted agent, or other radionuclide tracers have been applied in cancer and post-myocardial infarction neovessel imaging [142,143,144]. As MMPs participate in intraplaque angiogenesis, MMP inhibitor-based radiotracers bind to activated MMPs and detect angiogenic processes *in vivo*. Several inhibitors have been successfully labeled with ^123^I, ^11^C, or ^18^F for PET imaging, but no animal experiment currently exists [145,146]. Specific uptake of ^123^I-labelled MMP inhibitors into the plaques was shown in animals on a high-cholesterol diet by planar gamma camera, the radioactivity of which was 2.72-fold of the common artery [147]. Yet, direct visualization of VV neovascularization through PET or SPECT still remains unavailable. Autoradiography, another nuclear technique, can identify angiogenesis in atherosclerotic plaque *ex vivo* with ^125^I-labeled monoclonal antibodies against fibronectin extra-domain B (ED-B) [148]. More recently, ^99m^Tc-labeled membrane type 1 MMP monoclonal antibody (^99m^Tc-MT1-MMP mAb) accumulation, which detects active MMP-2 and MMP-13, was found in atheromatous lesions (4.8 ± 1.9, % injected dose × body weight/mm^2^ × 10^2^) and positively correlated with membrane type 1 MMP expression [149]. In conclusion, nuclear neovessel imaging could provide diagnostic imaging capability of vulnerable plaques, and further investigations to improve the modalities are strongly required.

Magnetic Resonance imaging (MRI), by virtue of its ability to characterize various pathological components of atherosclerosis plaques with advantages of high-resolution and radiation avoiding, is another promising modality for studying VV. On traditional anatomical dynamic contrast-enhanced MRI (DCE-MRI), the enhancement of the outer rim of the internal carotid arteries represents VV [150]. With the development of αvβ_3_ integrin-targeted gadolinium chelates, MRI molecular imaging emerged. With paramagnetic gadolinium-based nanoparticles, increased angiogenesis was detected as a 26% ± 4% and 47% ± 5% signal increase over baseline at 15 and 120 min in cholesterolemic rabbits, while only half of the signal augmentation was detected in cholesterol-fed rabbits that received non-targeted nanoparticles. A heterogeneous spatial distribution of neovessels was also observed through molecular imaging [151]. MRI with ανβ3-targeted nanoparticles also showed the ability to assess the therapeutic effect of anti-atherosclerotic agents. After 16 weeks of an appetite suppressant treatment with benfluorex, MR enhancement decreased in treated animals when a steady increase was seen in the untreated group [152]. Gadolinium quantitative MRI of VV reported that the total neovessel area in the matched sections from DCE-MRI correlated with the histological measurement with a high correlation coefficient of 0.80, thereby serving as a quantitative parameter in vascular imaging [153]. In humans, *in vivo* DCE-MRI showed that the transfer constant (K^trans^) of gadolinium enhancement in the carotid adventitia is a quantitative measurement of the VV extent, a method confirmed by histological measurements on a carotid endarterectomy specimen [154]. K^trans^ represents a kinetic parameter that characterizes the transfer of the contrast agent from plasma to the extravascular space (e.g., adventitia). The transfer therefore depends on VV density. By estimating K^trans^ via DCE-MRI, the transfer of gadolinium into the extravascular space, calculated from dynamic kinetics of tissue enhancement, correlates strongly with macrophage content, neovascularization, and loose matrix areas measured by histology analysis [155]. These studies indicated that adventitial enhancement seen on DCE-MRI with gadolinium chelates can be used to detect VV neovascularization in the process of atherosclerosis, therefore assessing the risk for plaque rupture. However, previous studies show gadolinium associates with nephrogenic systemic fibrosis in patients with reduced renal function [156]. Thus, exploration of more effective contrast enhancement agents with lower toxicity and higher resolution is needed in the future.

CEUS is another tool in molecular imaging. As discussed, CEUS has been employed not only to improve the evaluation of intima-media thickness, but also to highlight wall irregularities, ulcerations, adventitial VV and neovasculature of the atherosclerotic plaque that are often not visible by standard non-invasive ultrasound imaging [157]. Further development of conjugated microbubbles that bind to specific ligands in thrombotic material or neovessels has led to the term “molecular imaging” in CEUS scanning. Microbubbles coupled to the VEGFR may allow for a detection of neovascularization. Using dual ET-1/VEGFsp receptor (DEspR)-targeted microbubbles, CEUS molecular imaging has detected an increased DEspR-expression in carotid artery lesions and expanded VV neovessels in transgenic rats with carotid artery disease [158]. CEUS with VEGFR-2-targeted microbubbles was used to evaluate the response to sorafenib (a drug that inhibits cell proliferation and neovascularization in several tumors) in a mouse model of hepatocellular carcinoma [159]. The amount of bound microbubbles in the tumor quantified by dedicated software was lower in the treatment group through CEUS molecular imaging.

Together, as summarized in Table 1, molecular imaging shows an exciting potential in VV imaging by increasing the quality of resolution. Further studies are needed to modify the existing molecular imaging modalities and targeted agents that are safe, accurate, and easy to detect.

**Table 1:** Molecular imaging modalities and targets of vasa vasorum in atherosclerosis models.

Imaging Modality	Spatial Resolution	Temporal Resolution	Targets (Reference)	Species	*In Vivo* Imaging	Histological Validation	Results
Planar gamma camera	cm^3^	Hours	^123^I-labelled MMP inhibitors [147]	*Apoe^−/−^* mice	+	+	Signal of ^123^I-labelled inhibitors into plaques in high cholesterol animals was 2.72-folds of the control
Autoradiograph	μm^3^	Milliseconds	ED-B [148]	*Apoe^−/−^* mice	−	−	^125^I-labeled monoclonal antibodies against ED-B identified the angiogenesis in atherosclerotic plaques *ex vivo*
			99mTc-MT1-MMP mAb [149]	WHHLMI rabbits	+	+	The highest accumulation of ^99m^Tc-MT1-MMP mAb was found in atheromatous lesions in comparison with stable lesions
MRI	mm^3^	Seconds	Integrin αvβ_3_	Male New Zealand White (NZW) rabbits [151]	+	+	Paramagnetic gadolinium-based nanoparticles showed strong enhancement in atheroscletotic lesions that was twice of the non-targeted nanoparticles
				JCR:LA-cp rats [152]	+	+	The enhancement of ανβ3-targeted nanoparticles was preserved in benfluorex treating group
				Humans [153,154,155]	+	+	Targeted gadolinium compounds detected VV, total area and K^trans^ of the enhancement could be quantitative parameters
CEUS	μm^3^	Milliseconds	ET-1/VEGFsp receptor [158]	Tg25 (hCETP) Dahl-S rats	+	+	Expanded VV in transgenic rats with carotid artery disease were detected by targeted microbubbles
			VEGFR-2 [159]	Female nude mice	+	−	VEGFR-2 targeted microbubbles were able to evaluate anti-angiogenic effect of sorafenib

## 6. Anti- and Pro-Angiogenic Therapies on Vasa Vasorum

### 6.1. Anti-Angiogenic Therapies

The rupture of neovessels originating from VV may be the main cause of intraplaque hemorrhage in advanced atherosclerosis. It seems logical to speculate that anti-angiogenic strategies can be used to inhibit plaque growth and stabilize existing plaques, although lipid-controlling, anti-inflammation, and invasive angioplasty are among the current main treatments for atherosclerosis. As discussed above, angiogenic factors control neovascularization. Expression regulation of these factors (e.g., Ets-1, Ang-1 receptor, HIF-1α, MMPs) by microRNAs influences EC activation and SMC phenotype switch, thereby decreasing VV neovascularization [160]. In contrast, the effects of angiogenesis inhibitors on atherosclerotic lesions are difficult to classify because the various agents that block angiogenesis do not have uniformed mechanisms of action. Each agent may also have unique effects on different cell types and biochemical pathways that are also present in atherosclerotic lesions. To simplify the understanding of different angiogenic inhibitors, we grouped angiogenic inhibitors into three categories: direct anti-angiogenic molecules (*i.e.*, angiostatics) that are derived from protein proteolysis; inhibitors that target directly or indirectly the angiogenic factors (e.g., VEGF); and others with incompletely characterized mechanisms.

Direct anti-angiogenic compounds, also known as angiostatics, are substances that target ECs and SMCs without affecting endogenous angiogenic factors. Angiostatics were initially identified as tumor-derived factors that inhibit neovascularization of remote metastases of Lewis lung carcinoma (*i.e.*, angiostatin) [161] and hemangioendothelioma (*i.e.*, endostatin) [162]. Endostatin is a fragment from collagen-XVIII proteolysis. In apolipoprotein E-deficient (*Apoe^−/−^*) mice, chronic treatments with endostatin reduced intimal neovascularization and inhibited plaque growth by 85% in atherosclerosis [163]. However, as the plaque intimal SMC contents were similar between control and treated mice in this research, a mechanism for the anti-angiogenic property of endostatin remains incompletely understood. Angiostatin, a proteolytic fragment of plasminogen, blocks the angiogenic potential of atherosclerotic aortas with a parallel reduction of macrophages in the plaques and around VV [164]. Activated macrophages stimulate angiogenesis by recruiting more inflammatory cells to increase angiogenesis further. Angiostatin inhibition interrupts this positive feedback from inflammatory cells and hinders angiogenesis in atherosclerotic plaques. The mechanisms by which endostatin and angiostatin inhibit angiogenesis are not fully understood. Inhibition of EC and SMC proliferation and migration, without affecting endothelial intracellular signaling pathways, seems to play a vital role in this process [165].

Angiogenic factor inhibitors offer another target choice of anti-angiogenic therapy in atherosclerosis. The inhibition of the VEGF signaling pathway by targeting VEGF and VEGFR-1 has been well studied. Bevacizumab is a VEGF-specific antibody that has the capacity to inhibit VV after local delivery by stent in a rabbit atherosclerosis model [166]. Exogenous application of antibodies against VEGFR-1 reduced the size of early and intermediate plaques by 50% and the growth of advanced lesions by ~25% in *Apoe^−/−^* mice [167]. PlGF is a VEGFR-1 ligand. Deficiency of PlGF inhibited early-stage atherosclerosis. On the other hand, increased adventitial expression of PlGF promoted intimal hyperplasia and VV proliferation [168]. Importantly, the anti-angiogenic property of PlGF antibodies acted only on diseased arteries without affecting healthy vessels [169], offering a great advantage for patient treatment. Other angiogenic factor inhibitors include anti-MMP-2 and anti-MMP-9 antibodies that also have the ability to block endothelial cell tubule formation [170]. MicroRNAs that interfere with the expression of angiogenic factors (e.g., Ets-1, Ang-1 receptor, HIF-1α, MMPs) also decrease VV density in atherosclerotic plaques [160]. Apart from antibodies and microRNAs that act directly on angiogenic factors, indirect agents also play an anti-angiogenic role in VV angiogenesis. Thalidomide is an anti-angiogenic drug that exerts anti-inflammatory and immune-modulatory effects. Treatment with thalidomide preserved VV spatial density by 45% in high-cholesterol, diet-fed atherosclerosic pigs and was only 1.3-fold higher than that of normal pigs when compared with the 2.4-fold increase in the untreated group [171]. Reduced adventitial VV neovascularization and plaque progression after thalidomide treatment were also seen in *Apoe^−/−^/Ldlr^−/−^* double knockout mice [172]. The anti-angiogenic effect of thalidomide in atherosclerosis was accompanied by the inhibition of VEGF expression.

Another group of molecules remain difficult to classify because of their limited study and complicated mechanisms. ET-1 is a vasoconstrictor that has mitogenic activity on SMCs and participates in VV neovascularization during atherogenesis [173]. ET receptor antagonism (ET-A) application in hypercholesterolemic pigs showed that elevated VV density in the hypercholesterolemia group was greatly preserved by ~32% [174]. Fumagillin nanoparticles are also anti-angiogenic agents that target ECs. Treatment of atherosclerosic rabbits with αvβ_3_ integrin-targeted paramagnetic nanoparticles together with fumagillin decreased MRI enhancement and reduced the numbers of microvessels [175]. Anti-angiogenic rPAI-1_23_, a truncated isoform of plasminogen activator inhibitor-1 (PAI-1), greatly reduced VV, especially the second order VV, with a 37% reduction in total vessel area and a 43% reduction in vessel length in atherogenic female *ApoB-48^−/−^/Ldlr^−/−^* mice through inhibition of bFGF, suggesting a significant therapeutic potential of this anti-angiogenic protease inhibitor peptide in atherosclerosis [176]. Further study concluded that rPAI-1_23_ caused the regression of adventitial VV in hypercholesterolemic mice by increasing plasmin and MMP activities that degrade perlecan, nidogen, and fibrin in the extracellular milieu. Without a supportive scaffold from these extracellular matrix proteins, ECs may undergo apoptosis, leading to VV regression [177]. The anti-proliferative and anti-inflammatory drug, 3-deazaadenosine (c3Ado), dose-dependently prevents the proliferation and migration of human coronary artery ECs. It also inhibited VV neovascularization along the descending aortas in *Apoe^−/−^/Ldl^−/−^* double knockout mice [178]. Here we summarize this last category of molecules in Table 2.

**Table 2:** Anti-angiogenic molecules with incompletely characterized mechanisms.

Compounds	Year	Functions	Species	Possible Mechanisms	Results
ET-A	1993 [173], 2002 [174]	Inhibiting ET-1 receptor	Female domestic pigs	Inhibiting mitogenic activity of SMCs	Elevated VV density in hypercholesterolemia pigs were greatly preserved by ~32% after ET-A treatment
Fumagillin nanoparticle	2006 [175]	An anti-angiogenic agent that targets αvβ_3_ integrin	Male NZW rabbits	Not investigate	MRI enhancement and the numbers of microvessels are decreased in fumagillin-treated cholesterol-fed rabbits
rPAI-1_23_	2009 [176], 2011 [177]	A truncated isoform of plasminogen activator inhibitor-1 (PAI-1)	*Female ApoB-48^−/−^/Ldlr^−/−^* mice	Reducing FGF-2 expression. Increasing plasmin and MMP activities on degrading compounds (*i.e.*, perlecan, nidogen, fibrin) that produce supportive scaffold in the extracellular milieu, leading to apoptosis of ECs	A 37% reduction in total vessel area and a 43% reduction in vessel length of the second order VV are observed as a result of apoptosis of ECs
c3Ado	2009 [178]	An anti-proliferative and anti-inflammatory drug	*Apoe^−/−^/Ldl^−/−^* double knockout mice	Preventing the proliferation and migration of ECs	VV neovascularization is inhibited dose dependently

### 6.2. Pro-Angiogenic Therapies

Based on what has been discussed, anti-angiogenic therapies seem to be effective and significant in atherosclerosis medication, at least in animal models. However, a recent report from colorectal cancer patients noted that the anti-VEGF monoclonal antibody bevacizumab showed a higher risk (3.5 additional cases/1000 person-years) of arterial thromboembolic events (e.g., stroke, myocardial infarction, arterial embolism and thrombosis, and angina) despite its anti-angiogenic properties [179]. This observation put into question the risk of angiogenesis inhibitors to atherothrombosis and associated complications. It seems that the role of neovascularization is far more complicated than imagined. A case report from four patients suggested that VV are a source of collateral circulation after carotid artery occlusion secondary to atherosclerotic disease [180]. It has been demonstrated that VSMC-rich lesions are stable because of their high cellular content, whereas acellular lesions with a higher degree of calcification, fibrosis, and lipids are more prone to fracture or rupture [181]. It could be argued that, by enriching the supply of nutrients to the plaque core, neovascularization may increase plaque cellularity, thereby acting as an underlying cause of plaque stabilization.

Studies on HMG-CoA reductase inhibitors support the feasibility of pro-angiogenic therapies in atherosclerosis. Statins, such as atorvastatin and simvastatin, demonstrated beneficial effects in reducing atherosclerotic VV neovascularization independent of lipid lowering [129,182]. While high doses of cerivastatin (2.5 mg/kg/day) blocked angiogenesis, low doses of cerivastatin (0.5 mg/kg/day) induced angiogenesis *in vitro* [183]. In contrast, both high- and low-dose statin therapy blocked plaque progression, indicating that pro-angiogenic effects do not lead to pro-atherosclerotic effects, as we anticipated. The inhibition of VV neovascularization may not be the fundamental strategy for plaque stabilizing in atherosclerosis. More attention may be focused on the normalization and maturation of VV to reduce the risk of VV leakage. As a result, studies in pro-angiogenic therapies have been encouraged in recent years.

Nerve growth factor (NGF) is a potent angiogenic factor that is decreased in atherosclerosis-lesioned arteries [184]. NGF application increased the ratio of large matured vessels (≥20 μm in diameter) from 30% to ~50% compared with the control, whereas VEGF promoted more immature small microvessels (<20 μm) than the control. NGF also enhanced the maturation of VEGF-induced neovessels from 20% to 40% [185]. These studies suggested a therapeutic possibility of pro-angiogenic NGF on atherosclerosis by enhancing VV maturation. bFGF is another predominant angiogenic growth factor that was also required for VV plexus stability in hypercholesterolemic mice [186]. Although it was regarded as a pro-atherogenic factor due to its stimulatory activity on SMC growth [187], recent studies suggested a protective property of bFGF on plaque formation in a hypercholesterolemic rabbit model by reversing endothelial dysfunction and reducing vascular cell adhesion molecule-1 (VCAM-1) expression as well as plaque macrophage content [188]. However, increased endothelial FGF-receptor-2 signaling by EC-selective overexpression of FGF-receptor-2 aggravated atherosclerosis by promoting p21^Cip1^-mediated EC dysfunction [189]. Therefore the use of bFGF for therapeutic angiogenesis such as ischemic injury may have to be considered due to the possible adverse effects in aggravating atherosclerosis. For example, simvastatin with 1.8 mg/kg/day in rabbits increased the expression of HIF-1α and VEGFR-2 in advanced peri-infarcted myocardium, but blocked the protein expression of HIF-1α, VEGF, and VEGFR-2 in early atherosclerotic arteries. Apart from the dosage of this statin, the stage of atherosclerotic disease may influence the outcome of this statin therapy [190]. Therefore, the stage of atherosclerosis and the microenvironment of plaque should be considered to determine whether a pro-angiogenesis or anti-angiogenesis therapy should be employed.

In conclusion, the role of neovescularization in plaque rupture cannot be simply defined. It is instead an intricate process that may exert various physiological effects in different stages of atherosclerosis. Pro-angiogenic factors that improve the maturation and stabilization of VV and reduce the leakage of these neovessels seem essential in further anti-atherosclerosis studies. The stage of plaque development may be a major determinant of whether we should employ anti-angiogenesis or pro-angiogenesis approaches.

## 7. Conclusions

VV neovascularization exists in the process of atherosclerosis, which seems like a compensatory reaction in order to provide adequate oxygen and nourishment for atherosclerotic arteries. However, the imbalance between angiogenic and anti-angiogenic factors along with latter damages leads to the dysfunction of ECs and their surrounding supporting cells, resulting in an immature and fragile VV neovasculature with weak integrity. In addition to the VV leakage itself, further stimulation of inflammation and necrosis of atherosclerotic plaques is another cause of VV-triggered intraplaque hemorrhage. It remains unclear if VV play a causative or reactive role in the atherosclerotic process, yet VV assessment has been suspected as an effective marker in the early detection of vulnerable plaques.

Several imaging modalities have been established to visualize VV neovascularization and to identify plaques with a high risk of rupture. Anatomical imaging techniques such as IVUS and OCT provide high-resolution images of VV and atherosclerotic plaques yet fail to recognize the biological features of the tissue. Recent molecular imaging modalities exhibit both the anatomy and pathological function of VV, which benefit from the development of specific target agents. Molecular imaging methods such as DCE-MRI and CEUS are extensively applied in visualizing VV. The results of these studies are encouraging, but problems remain. The toxicity of enhancement agents, the limited resolution of imaging, and some controversial results from different studies all lead to the need of improving the existing imaging modalities and investigating effective, and practical methods that are safe and precise for bedside use.

Considering the role of VV neovascularization in the process of atherosclerosis, anti-angiogenic therapies seem logical to prevent or attenuate the deterioration of this disease. Several studies have proven that angiogenesis inhibitors preserve the density of intraplaque VV in atherosclerosis. However, a higher risk of arterial cardiovascular events is observed in colorectal cancer patients using angiogenic inhibitors. The use of anti-angiogenic therapy as a proper way to treat atherosclerosis and prevent intraplaque hemorrhage remains complicated. The application of pro-angiogenic NGF and bFGF showed the capacity of enhancing the maturation of VV neovessels. Therefore, pro-angiogenesis factors that improve the maturation of VV and reduce the leakage of these neovessels may be the fundamental solution in reducing intraplaque hemorrhage, and may lead to a new direction for the future study of stabilizing vulnerable plaques.

## References

[1] Mozaffarian D., Benjamin E.J., Go A.S., Arnett D.K., Blaha M.J., Cushman M., de Ferranti S., Despres J.P., Fullerton H.J., Howard V.J. (2015). Heart disease and stroke statistics—2015 update: A report from the American Heart Association. Circulation.

[2] Davies M.J., Richardson P.D., Woolf N., Katz D.R., Mann J. (1993). Risk of thrombosis in human atherosclerotic plaques: Role of extracellular lipid, macrophage, and smooth muscle cell content. Br. Heart J..

[3] De Boer O.J., van der Wal A.C., Teeling P., Becker A.E. (1999). Leucocyte recruitment in rupture prone regions of lipid-rich plaques: A prominent role for neovascularization?. Cardiovasc. Res..

[4] Staub D., Patel M.B., Tibrewala A., Ludden D., Johnson M., Espinosa P., Coll B., Jaeger K.A., Feinstein S.B. (2010). Vasa vasorum and plaque neovascularization on contrast-enhanced carotid ultrasound imaging correlates with cardiovascular disease and past cardiovascular events. Stroke J. Cereb. Circ..

[5] Takano M., Mizuno K., Okamatsu K., Yokoyama S., Ohba T., Sakai S. (2001). Mechanical and structural characteristics of vulnerable plaques: Analysis by coronary angioscopy and intravascular ultrasound. J. Am. Coll. Cardiol..

[6] Ritman E.L., Lerman A. (2007). The dynamic vasa vasorum. Cardiovasc. Res..

[7] Koester W. (1876). Endareritis and arteritis. Berl. Klin. Wochenschr..

[8] Paterson J.C. (1936). Vascularization and hemorrhage of the intima of arteriosclerotic coronary arteries. Arch. Pathol..

[9] Patterson J.C. (1938). Capillary rupture with intimal hemorrhage as a causative factor in coronary thrombosis. Arch. Pathol..

[10] Barger A.C., Beeuwkes R., Lainey L.L., Silverman K.J. (1984). Hypothesis: Vasa vasorum and neovascularization of human coronary arteries. A possible role in the pathophysiology of atherosclerosis. N. Engl. J. Med..

[11] Gossl M., Versari D., Hildebrandt H.A., Bajanowski T., Sangiorgi G., Erbel R., Ritman E.L., Lerman L.O., Lerman A. (2010). Segmental heterogeneity of vasa vasorum neovascularization in human coronary atherosclerosis. JACC Cardiovasc. Imaging.

[12] Kwon H.M., Sangiorgi G., Ritman E.L., McKenna C., Holmes D.R., Schwartz R.S., Lerman A. (1998). Enhanced coronary vasa vasorum neovascularization in experimental hypercholesterolemia. J. Clin. Investig..

[13] Dunmore B.J., McCarthy M.J., Naylor A.R., Brindle N.P. (2007). Carotid plaque instability and ischemic symptoms are linked to immaturity of microvessels within plaques. J. Vasc. Surg..

[14] Sluimer J.C., Kolodgie F.D., Bijnens A.P., Maxfield K., Pacheco E., Kutys B., Duimel H., Frederik P.M., van Hinsbergh V.W., Virmani R. (2009). Thin-walled microvessels in human coronary atherosclerotic plaques show incomplete endothelial junctions relevance of compromised structural integrity for intraplaque microvascular leakage. J. Am. Coll. Cardiol..

[15] Khurana R., Zhuang Z., Bhardwaj S., Murakami M., de Muinck E., Yla-Herttuala S., Ferrara N., Martin J.F., Zachary I., Simons M. (2004). Angiogenesis-dependent and independent phases of intimal hyperplasia. Circulation.

[16] Herrmann J., Lerman L.O., Rodriguez-Porcel M., Holmes D.R., Richardson D.M., Ritman E.L., Lerman A. (2001). Coronary vasa vasorum neovascularization precedes epicardial endothelial dysfunction in experimental hypercholesterolemia. Cardiovasc. Res..

[17] Maiellaro K., Taylor W.R. (2007). The role of the adventitia in vascular inflammation. Cardiovasc. Res..

[18] Clarke J.A. (1966). An X-ray microscopic study of the postnatal development of the vasa vasorum of normal human coronary arteries. Acta Anat..

[19] Wolinsky H., Glagov S. (1967). Nature of species differences in the medial distribution of aortic vasa vasorum in mammals. Circ. Res..

[20] Schoenenberger F., Mueller A. (1960). On the vascularization of the bovine aortic wall. Helv. Physiol. Pharmacol. Acta.

[21] Gossl M., Rosol M., Malyar N.M., Fitzpatrick L.A., Beighley P.E., Zamir M., Ritman E.L. (2003). Functional anatomy and hemodynamic characteristics of vasa vasorum in the walls of porcine coronary arteries. Anat. Rec. Part A Discov. Mol. Cell. Evol. Biol..

[22] Fleiner M., Kummer M., Mirlacher M., Sauter G., Cathomas G., Krapf R., Biedermann B.C. (2004). Arterial neovascularization and inflammation in vulnerable patients: Early and late signs of symptomatic atherosclerosis. Circulation.

[23] Bitar R., Moody A.R., Leung G., Symons S., Crisp S., Butany J., Rowsell C., Kiss A., Nelson A., Maggisano R. (2008). *In vivo* 3D high-spatial-resolution MR imaging of intraplaque hemorrhage. Radiology.

[24] Acoltzin Vidal C., Maldonado Villasenor I., Rodriguez Cisneros L., Muniz Murguia J.J. (2004). Diminished vascular density in the aortic wall. Morphological and functional characteristics of atherosclerosis. Arch. Cardiol. Mexico.

[25] Rademakers T., Douma K., Hackeng T.M., Post M.J., Sluimer J.C., Daemen M.J., Biessen E.A., Heeneman S., van Zandvoort M.A. (2013). Plaque-associated vasa vasorum in aged apolipoprotein E-deficient mice exhibit proatherogenic functional features *in vivo*. Arterioscler. Thromb. Vasc. Biol..

[26] Gossl M., Malyar N.M., Rosol M., Beighley P.E., Ritman E.L. (2003). Impact of coronary vasa vasorum functional structure on coronary vessel wall perfusion distribution. Am. J. Physiol. Heart Circ. Physiol..

[27] Han D.G. (2010). The innateness of coronary artery: Vasa vasorum. Med. Hypotheses.

[28] Moss A.J., Samuelson P., Angell C., Minken S.L. (1968). Polarographic evaluation of transmural oxygen availabitlity in intact muscular arteries. J. Atheroscler. Res..

[29] Crawford D.W., Back L.H., Cole M.A. (1980). *In vivo* oxygen transport in the normal rabbit femoral arterial wall. J. Clin. Investig..

[30] Bratzler R.L., Chisolm G.M., Colton C.K., Smith K.A., Lees R.S. (1977). The distribution of labeled low-density lipoproteins across the rabbit thoracic aorta *in vivo*. Atherosclerosis.

[31] Scotland R.S., Vallance P.J., Ahluwalia A. (2000). Endogenous factors involved in regulation of tone of arterial vasa vasorum: Implications for conduit vessel physiology. Cardiovasc. Res..

[32] Scotland R., Vallance P., Ahluwalia A. (1999). Endothelin alters the reactivity of vasa vasorum: Mechanisms and implications for conduit vessel physiology and pathophysiology. Br. J. Pharmacol..

[33] Ohhira A., Ohhashi T. (1992). Effects of aortic pressure and vasoactive agents on the vascular resistance of the vasa vasorum in canine isolated thoracic aorta. J. Physiol..

[34] Vio A., Gozzetti G., Reggiani A. (1967). Importance of the vasa vasorum in the healing processes of arterial sutures. (Experimental study on the dog). Boll. Soc. Ital. Biol. Sper..

[35] Winternitz M.C., Thomas R.M., LeCompte P.M. (1938). The Biology of Arteriosclerosis.

[36] Ribatti D., Levi-Schaffer F., Kovanen P.T. (2008). Inflammatory angiogenesis in atherogenesis—A double-edged sword. Ann. Med..

[37] Williams J.K., Armstrong M.L., Heistad D.D. (1988). Vasa vasorum in atherosclerotic coronary arteries: Responses to vasoactive stimuli and regression of atherosclerosis. Circ. Res..

[38] Sano M., Sasaki T., Hirakawa S., Sakabe J., Ogawa M., Baba S., Zaima N., Tanaka H., Inuzuka K., Yamamoto N. (2014). Lymphangiogenesis and angiogenesis in abdominal aortic aneurysm. PLoS ONE.

[39] Davie N.J., Gerasimovskaya E.V., Hofmeister S.E., Richman A.P., Jones P.L., Reeves J.T., Stenmark K.R. (2006). Pulmonary artery adventitial fibroblasts cooperate with vasa vasorum endothelial cells to regulate vasa vasorum neovascularization: A process mediated by hypoxia and endothelin-1. Am. J. Pathol..

[40] Bjornheden T., Levin M., Evaldsson M., Wiklund O. (1999). Evidence of hypoxic areas within the arterial wall *in vivo*. Arterioscler. Thromb. Vasc. Biol..

[41] Nakashima Y., Chen Y.X., Kinukawa N., Sueishi K. (2002). Distributions of diffuse intimal thickening in human arteries: Preferential expression in atherosclerosis-prone arteries from an early age. Virchows Arch. Int. J. Pathol..

[42] Zemplenyi T., Crawford D.W., Cole M.A. (1989). Adaptation to arterial wall hypoxia demonstrated *in vivo* with oxygen microcathodes. Atherosclerosis.

[43] Jarvilehto M., Tuohimaa P. (2009). Vasa vasorum hypoxia: Initiation of atherosclerosis. Med. Hypotheses.

[44] Den Hartog J.P. (1949). Strength of Materials.

[45] Bjornheden T., Bondjers G. (1987). Oxygen consumption in aortic tissue from rabbits with diet-induced atherosclerosis. Arteriosclerosis.

[46] Sluimer J.C., Gasc J.M., van Wanroij J.L., Kisters N., Groeneweg M., Sollewijn Gelpke M.D., Cleutjens J.P., van den Akker L.H., Corvol P., Wouters B.G. (2008). Hypoxia, hypoxia-inducible transcription factor, and macrophages in human atherosclerotic plaques are correlated with intraplaque angiogenesis. J. Am. Coll. Cardiol..

[47] Kwon H.M., Sangiorgi G., Ritman E.L., Lerman A., McKenna C., Virmani R., Edwards W.D., Holmes D.R., Schwartz R.S. (1998). Adventitial vasa vasorum in balloon-injured coronary arteries: Visualization and quantitation by a microscopic three-dimensional computed tomography technique. J. Am. Coll. Cardiol..

[48] Gossl M., Versari D., Mannheim D., Ritman E.L., Lerman L.O., Lerman A. (2007). Increased spatial vasa vasorum density in the proximal LAD in hypercholesterolemia—Implications for vulnerable plaque-development. Atherosclerosis.

[49] Sun Z. (2014). Atherosclerosis and atheroma plaque rupture: Imaging modalities in the visualization of vasa vasorum and atherosclerotic plaques. Sci. World J..

[50] Gossl M., Versari D., Lerman L.O., Chade A.R., Beighley P.E., Erbel R., Ritman E.L. (2009). Low vasa vasorum densities correlate with inflammation and subintimal thickening: Potential role in location--determination of atherogenesis. Atherosclerosis.

[51] Lim C.S., Kiriakidis S., Sandison A., Paleolog E.M., Davies A.H. (2013). Hypoxia-inducible factor pathway and diseases of the vascular wall. J. Vasc. Surg..

[52] Semenza G.L., Agani F., Booth G., Forsythe J., Iyer N., Jiang B.H., Leung S., Roe R., Wiener C., Yu A. (1997). Structural and functional analysis of hypoxia-inducible factor 1. Kidney Int..

[53] Oikawa M., Abe M., Kurosawa H., Hida W., Shirato K., Sato Y. (2001). Hypoxia induces transcription factor ETS-1 via the activity of hypoxia-inducible factor-1. Biochem. Biophys. Res. Commun..

[54] Leung D.W., Cachianes G., Kuang W.J., Goeddel D.V., Ferrara N. (1989). Vascular endothelial growth factor is a secreted angiogenic mitogen. Science.

[55] Ushio-Fukai M. (2007). VEGF signaling through NADPH oxidase-derived ROS. Antioxid. Redox Signal..

[56] Shweiki D., Itin A., Soffer D., Keshet E. (1992). Vascular endothelial growth factor induced by hypoxia may mediate hypoxia-initiated angiogenesis. Nature.

[57] Kuwahara F., Kai H., Tokuda K., Shibata R., Kusaba K., Tahara N., Niiyama H., Nagata T., Imaizumi T. (2002). Hypoxia-inducible factor-1α/vascular endothelial growth factor pathway for adventitial vasa vasorum formation in hypertensive rat aorta. Hypertension.

[58] Joukov V., Pajusola K., Kaipainen A., Chilov D., Lahtinen I., Kukk E., Saksela O., Kalkkinen N., Alitalo K. (1996). A novel vascular endothelial growth factor, VEGF-C, is a ligand for the Flt4 (VEGFR-3) and KDR (VEGFR-2) receptor tyrosine kinases. EMBO J..

[59] Achen M.G., Jeltsch M., Kukk E., Makinen T., Vitali A., Wilks A.F., Alitalo K., Stacker S.A. (1998). Vascular endothelial growth factor D (VEGF-D) is a ligand for the tyrosine kinases VEGF receptor 2 (Flk1) and VEGF receptor 3 (Flt4). Proc. Natl. Acad. Sci. USA.

[60] Kaipainen A., Korhonen J., Mustonen T., van Hinsbergh V.W., Fang G.H., Dumont D., Breitman M., Alitalo K. (1995). Expression of the fms-like tyrosine kinase 4 gene becomes restricted to lymphatic endothelium during development. Proc. Natl. Acad. Sci. USA.

[61] Kukk E., Lymboussaki A., Taira S., Kaipainen A., Jeltsch M., Joukov V., Alitalo K. (1996). VEGF-C receptor binding and pattern of expression with VEGFR-3 suggests a role in lymphatic vascular development. Development.

[62] Partanen T.A., Arola J., Saaristo A., Jussila L., Ora A., Miettinen M., Stacker S.A., Achen M.G., Alitalo K. (2000). VEGF-C and VEGF-D expression in neuroendocrine cells and their receptor, VEGFR-3, in fenestrated blood vessels in human tissues. FASEB J..

[63] Rutanen J., Leppanen P., Tuomisto T.T., Rissanen T.T., Hiltunen M.O., Vajanto I., Niemi M., Hakkinen T., Karkola K., Stacker S.A. (2003). Vascular endothelial growth factor-D expression in human atherosclerotic lesions. Cardiovasc. Res..

[64] Belgore F., Blann A., Neil D., Ahmed A.S., Lip G.Y. (2004). Localisation of members of the vascular endothelial growth factor (VEGF) family and their receptors in human atherosclerotic arteries. J. Clin. Pathol..

[65] Vuorio T., Nurmi H., Moulton K., Kurkipuro J., Robciuc M.R., Ohman M., Heinonen S.E., Samaranayake H., Heikura T., Alitalo K. (2014). Lymphatic vessel insufficiency in hypercholesterolemic mice alters lipoprotein levels and promotes atherogenesis. Arterioscler. Thromb. Vasc. Biol..

[66] Iwasaka C., Tanaka K., Abe M., Sato Y. (1996). Ets-1 regulates angiogenesis by inducing the expression of urokinase-type plasminogen activator and matrix metalloproteinase-1 and the migration of vascular endothelial cells. J. Cell. Physiol..

[67] Hashiya N., Jo N., Aoki M., Matsumoto K., Nakamura T., Sato Y., Ogata N., Ogihara T., Kaneda Y., Morishita R. (2004). In vivo evidence of angiogenesis induced by transcription factor Ets-1: Ets-1 is located upstream of angiogenesis cascade. Circulation.

[68] Higashida T., Kanno H., Nakano M., Funakoshi K., Yamamoto I. (2008). Expression of hypoxia-inducible angiogenic proteins (hypoxia-inducible factor-1α, vascular endothelial growth factor, and E26 transformation-specific-1) and plaque hemorrhage in human carotid atherosclerosis. J. Neurosurg..

[69] Kitange G., Shibata S., Tokunaga Y., Yagi N., Yasunaga A., Kishikawa M., Naito S. (1999). Ets-1 transcription factor-mediated urokinase-type plasminogen activator expression and invasion in glioma cells stimulated by serum and basic fibroblast growth factors. Lab. Investig. J. Tech. Methods Pathol..

[70] Paumelle R., Tulasne D., Kherrouche Z., Plaza S., Leroy C., Reveneau S., Vandenbunder B., Fafeur V. (2002). Hepatocyte growth factor/scatter factor activates the ETS1 transcription factor by a RAS-RAF-MEK-ERK signaling pathway. Oncogene.

[71] Langheinrich A.C., Kampschulte M., Scheiter F., Dierkes C., Stieger P., Bohle R.M., Weidner W. (2010). Atherosclerosis, inflammation and lipoprotein glomerulopathy in kidneys of apoE^−/−^/LDL^−/−^ double knockout mice. BMC Nephrol..

[72] Kumamoto M., Nakashima Y., Sueishi K. (1995). Intimal neovascularization in human coronary atherosclerosis: Its origin and pathophysiological significance. Hum. Pathol..

[73] Yamashita A., Shoji K., Tsuruda T., Furukoji E., Takahashi M., Nishihira K., Tamura S., Asada Y. (2008). Medial and adventitial macrophages are associated with expansive atherosclerotic remodeling in rabbit femoral artery. Histol. Histopathol..

[74] Sluimer J.C., Daemen M.J. (2009). Novel concepts in atherogenesis: Angiogenesis and hypoxia in atherosclerosis. J. Pathol..

[75] Brown A.J., Dean R.T., Jessup W. (1996). Free and esterified oxysterol: Formation during copper-oxidation of low density lipoprotein and uptake by macrophages. J. Lipid Res..

[76] Ho-Tin-Noe B., le Dall J., Gomez D., Louedec L., Vranckx R., El-Bouchtaoui M., Legres L., Meilhac O., Michel J.B. (2011). Early atheroma-derived agonists of peroxisome proliferator-activated receptor-gamma trigger intramedial angiogenesis in a smooth muscle cell-dependent manner. Circ. Res..

[77] Sahebkar A., Watts G.F. (2013). New LDL-cholesterol lowering therapies: Pharmacology, clinical trials, and relevance to acute coronary syndromes. Clin. Ther..

[78] Lusis A.J. (2000). Atherosclerosis. Nature.

[79] Kolodgie F.D., Gold H.K., Burke A.P., Fowler D.R., Kruth H.S., Weber D.K., Farb A., Guerrero L.J., Hayase M., Kutys R. (2003). Intraplaque hemorrhage and progression of coronary atheroma. N. Engl. J. Med..

[80] Tanaka K., Nagata D., Hirata Y., Tabata Y., Nagai R., Sata M. (2011). Augmented angiogenesis in adventitia promotes growth of atherosclerotic plaque in apolipoprotein E-deficient mice. Atherosclerosis.

[81] Moulton K.S. (2006). Angiogenesis in atherosclerosis: Gathering evidence beyond speculation. Curr. Opin. Lipidol..

[82] Ricote M., Li A.C., Willson T.M., Kelly C.J., Glass C.K. (1998). The peroxisome proliferator-activated receptor-gamma is a negative regulator of macrophage activation. Nature.

[83] Fang L., Liu C., Miller Y.I. (2014). Zebrafish models of dyslipidemia: Relevance to atherosclerosis and angiogenesis. Transl. Res. J. Lab. Clin. Med..

[84] Salomon R.G., Hong L., Hollyfield J.G. (2011). Discovery of carboxyethylpyrroles (CEPs): Critical insights into AMD, autism, cancer, and wound healing from basic research on the chemistry of oxidized phospholipids. Chem. Res. Toxicol..

[85] West X.Z., Malinin N.L., Merkulova A.A., Tischenko M., Kerr B.A., Borden E.C., Podrez E.A., Salomon R.G., Byzova T.V. (2010). Oxidative stress induces angiogenesis by activating TLR2 with novel endogenous ligands. Nature.

[86] Chatterjee T.K., Stoll L.L., Denning G.M., Harrelson A., Blomkalns A.L., Idelman G., Rothenberg F.G., Neltner B., Romig-Martin S.A., Dickson E.W. (2009). Proinflammatory phenotype of perivascular adipocytes: Influence of high-fat feeding. Circ. Res..

[87] Barandier C., Montani J.P., Yang Z. (2005). Mature adipocytes and perivascular adipose tissue stimulate vascular smooth muscle cell proliferation: Effects of aging and obesity. Am. J. Physiol. Heart Circ. Physiol..

[88] Wang P., Xu T.Y., Guan Y.F., Su D.F., Fan G.R., Miao C.Y. (2009). Perivascular adipose tissue-derived visfatin is a vascular smooth muscle cell growth factor: Role of nicotinamide mononucleotide. Cardiovasc. Res..

[89] Manka D., Chatterjee T.K., Stoll L.L., Basford J.E., Konaniah E.S., Srinivasan R., Bogdanov V.Y., Tang Y., Blomkalns A.L., Hui D.Y., Weintraub N.L. (2014). Transplanted perivascular adipose tissue accelerates injury-induced neointimal hyperplasia: Role of monocyte chemoattractant protein-1. Arterioscler. Thromb. Vasc. Biol..

[90] Rajsheker S., Manka D., Blomkalns A.L., Chatterjee T.K., Stoll L.L., Weintraub N.L. (2010). Crosstalk between perivascular adipose tissue and blood vessels. Curr. Opin. Pharmacol..

[91] Garcia-Touchard A., Henry T.D., Sangiorgi G., Spagnoli L.G., Mauriello A., Conover C., Schwartz R.S. (2005). Extracellular proteases in atherosclerosis and restenosis. Arterioscler. Thromb. Vasc. Biol..

[92] Liu X.Q., Mao Y., Wang B., Lu X.T., Bai W.W., Sun Y.Y., Liu Y., Liu H.M., Zhang L., Zhao Y.X. (2014). Specific matrix metalloproteinases play different roles in intraplaque angiogenesis and plaque instability in rabbits. PLoS ONE.

[93] Svensson P.A., Olson F.J., Hagg D.A., Ryndel M., Wiklund O., Karlstrom L., Hulthe J., Carlsson L.M., Fagerberg B. (2008). Urokinase-type plasminogen activator receptor is associated with macrophages and plaque rupture in symptomatic carotid atherosclerosis. Int. J. Mol. Med..

[94] Mattock K.L., Gough P.J., Humphries J., Burnand K., Patel L., Suckling K.E., Cuello F., Watts C., Gautel M., Avkiran M. (2010). Legumain and cathepsin-L expression in human unstable carotid plaque. Atherosclerosis.

[95] Li W., Kornmark L., Jonasson L., Forssell C., Yuan X.M. (2009). Cathepsin L is significantly associated with apoptosis and plaque destabilization in human atherosclerosis. Atherosclerosis.

[96] Le Dall J., Ho-Tin-Noe B., Louedec L., Meilhac O., Roncal C., Carmeliet P., Germain S., Michel J.B., Houard X. (2010). Immaturity of microvessels in haemorrhagic plaques is associated with proteolytic degradation of angiogenic factors. Cardiovasc. Res..

[97] Leclercq A., Houard X., Philippe M., Ollivier V., Sebbag U., Meilhac O., Michel J.B. (2007). Involvement of intraplaque hemorrhage in atherothrombosis evolution via neutrophil protease enrichment. J. Leukoc. Biol..

[98] Maisonpierre P.C., Suri C., Jones P.F., Bartunkova S., Wiegand S.J., Radziejewski C., Compton D., McClain J., Aldrich T.H., Papadopoulos N. (1997). Angiopoietin-2, a natural antagonist for Tie2 that disrupts *in vivo* angiogenesis. Science.

[99] Hashimoto T., Lam T., Boudreau N.J., Bollen A.W., Lawton M.T., Young W.L. (2001). Abnormal balance in the angiopoietin-tie2 system in human brain arteriovenous malformations. Circ. Res..

[100] Anagnostopoulos A., Eleftherakis-Papaiakovou V., Kastritis E., Tsionos K., Bamias A., Meletis J., Dimopoulos M.A., Terpos E. (2007). Serum concentrations of angiogenic cytokines in Waldenstrom macroglobulinaemia: The ration of angiopoietin-1 to angiopoietin-2 and angiogenin correlate with disease severity. Br. J. Haematol..

[101] Post S., Peeters W., Busser E., Lamers D., Sluijter J.P., Goumans M.J., de Weger R.A., Moll F.L., Doevendans P.A., Pasterkamp G. (2008). Balance between angiopoietin-1 and angiopoietin-2 is in favor of angiopoietin-2 in atherosclerotic plaques with high microvessel density. J. Vasc. Res..

[102] Chistiakov D.A., Orekhov A.N., Bobryshev Y.V. (2015). Contribution of neovascularization and intraplaque haemorrhage to atherosclerotic plaque progression and instability. Acta Physiol..

[103] Nagy E., Eaton J.W., Jeney V., Soares M.P., Varga Z., Galajda Z., Szentmiklosi J., Mehes G., Csonka T., Smith A. (2010). Red cells, hemoglobin, heme, iron, and atherogenesis. Arterioscler. Thromb. Vasc. Biol..

[104] Balla J., Jacob H.S., Balla G., Nath K., Eaton J.W., Vercellotti G.M. (1993). Endothelial-cell heme uptake from heme proteins: Induction of sensitization and desensitization to oxidant damage. Proc. Natl. Acad. Sci. USA.

[105] Potor L., Banyai E., Becs G., Soares M.P., Balla G., Balla J., Jeney V. (2013). Atherogenesis may involve the prooxidant and proinflammatory effects of ferryl hemoglobin. Oxid. Med. Cell. Longev..

[106] Juckett M.B., Balla J., Balla G., Jessurun J., Jacob H.S., Vercellotti G.M. (1995). Ferritin protects endothelial cells from oxidized low density lipoprotein *in vitro*. Am. J. Pathol..

[107] Lee F.Y., Lee T.S., Pan C.C., Huang A.L., Chau L.Y. (1998). Colocalization of iron and ceroid in human atherosclerotic lesions. Atherosclerosis.

[108] Baldwin A.L. (1999). Modified hemoglobins produce venular interendothelial gaps and albumin leakage in the rat mesentery. Am. J. Physiol..

[109] Abela G.S., Aziz K., Vedre A., Pathak D.R., Talbott J.D., Dejong J. (2009). Effect of cholesterol crystals on plaques and intima in arteries of patients with acute coronary and cerebrovascular syndromes. Am. J. Cardiol..

[110] Kaartinen M., Penttila A., Kovanen P.T. (1996). Mast cells accompany microvessels in human coronary atheromas: Implications for intimal neovascularization and hemorrhage. Atherosclerosis.

[111] Ambrose J.A., Tannenbaum M.A., Alexopoulos D., Hjemdahl-Monsen C.E., Leavy J., Weiss M., Borrico S., Gorlin R., Fuster V. (1988). Angiographic progression of coronary artery disease and the development of myocardial infarction. J. Am. Coll. Cardiol..

[112] Hyafil F., Cornily J.C., Feig J.E., Gordon R., Vucic E., Amirbekian V., Fisher E.A., Fuster V., Feldman L.J., Fayad Z.A. (2007). Noninvasive detection of macrophages using a nanoparticulate contrast agent for computed tomography. Nat. Med..

[113] Romero J.M., Pizzolato R., Atkinson W., Meader A., Jaimes C., Lamuraglia G., Jaff M.R., Buonanno F., Delgado Almandoz J., Gonzalez R.G. (2013). Vasa vasorum enhancement on computerized tomographic angiography correlates with symptomatic patients with 50% to 70% carotid artery stenosis. Stroke J. Cereb. Circ..

[114] Sadeghi M.M., Glover D.K., Lanza G.M., Fayad Z.A., Johnson L.L. (2010). Imaging atherosclerosis and vulnerable plaque. J. Nucl. Med..

[115] Li W., van der Steen A.F., Lancee C.T., Cespedes I., Bom N. (1998). Blood flow imaging and volume flow quantitation with intravascular ultrasound. Ultrasound Med. Biol..

[116] Moritz R., Eaker D.R., Anderson J.L., Kline T.L., Jorgensen S.M., Lerman A., Ritman E.L. (2012). IVUS detection of vasa vasorum blood flow distribution in coronary artery vessel wall. JACC Cardiovasc. Imaging.

[117] Papaioannou T.G., Vavuranakis M., Androulakis A., Lazaros G., Kakadiaris I., Vlaseros I., Naghavi M., Kallikazaros I., Stefanadis C. (2009). *In-vivo* imaging of carotid plaque neoangiogenesis with contrast-enhanced harmonic ultrasound. Int. J. Cardiol..

[118] O’Malley S.M., Vavuranakis M., Naghavi M., Kakadiaris I.A. (2005). Intravascular ultrasound-based imaging of vasa vasorum for the detection of vulnerable atherosclerotic plaque. Med. Image Comput. Comput. Assist. Interv. MICCAI.

[119] Vavuranakis M., Kakadiaris I.A., O’Malley S.M., Papaioannou T.G., Sanidas E.A., Naghavi M., Carlier S., Tousoulis D., Stefanadis C. (2008). A new method for assessment of plaque vulnerability based on vasa vasorum imaging, by using contrast-enhanced intravascular ultrasound and differential image analysis. Int. J. Cardiol..

[120] Goertz D.E., Frijlink M.E., Tempel D., van Damme L.C., Krams R., Schaar J.A., Ten Cate F.J., Serruys P.W., de Jong N., van der Steen A.F. (2006). Contrast harmonic intravascular ultrasound: A feasibility study for vasa vasorum imaging. Investig. Radiol..

[121] Goertz D.E., Frijlink M.E., Tempel D., Bhagwandas V., Gisolf A., Krams R., de Jong N., van der Steen A.F. (2007). Subharmonic contrast intravascular ultrasound for vasa vasorum imaging. Ultrasound Med. Biol..

[122] Goertz D.E., Frijlink M.E., de Jong N., van der Steen A.F. (2006). Nonlinear intravascular ultrasound contrast imaging. Ultrasound Med. Biol..

[123] Magnoni M., Coli S., Marrocco-Trischitta M.M., Melisurgo G., de Dominicis D., Cianflone D., Chiesa R., Feinstein S.B., Maseri A. (2009). Contrast-enhanced ultrasound imaging of periadventitial vasa vasorum in human carotid arteries. Eur. J. Echocardiogr..

[124] Shah F., Balan P., Weinberg M., Reddy V., Neems R., Feinstein M., Dainauskas J., Meyer P., Goldin M., Feinstein S.B. (2007). Contrast-enhanced ultrasound imaging of atherosclerotic carotid plaque neovascularization: A new surrogate marker of atherosclerosis?. Vasc. Med..

[125] Schinkel A.F., Krueger C.G., Tellez A., Granada J.F., Reed J.D., Hall A., Zang W., Owens C., Kaluza G.L., Staub D. (2010). Contrast-enhanced ultrasound for imaging vasa vasorum: Comparison with histopathology in a swine model of atherosclerosis. Eur. J. Echocardiogr..

[126] Coli S., Magnoni M., Sangiorgi G., Marrocco-Trischitta M.M., Melisurgo G., Mauriello A., Spagnoli L., Chiesa R., Cianflone D., Maseri A. (2008). Contrast-enhanced ultrasound imaging of intraplaque neovascularization in carotid arteries: Correlation with histology and plaque echogenicity. J. Am. Coll. Cardiol..

[127] Lee S.C., Carr C.L., Davidson B.P., Ellegala D., Xie A., Ammi A., Belcik T., Lindner J.R. (2010). Temporal characterization of the functional density of the vasa vasorum by contrast-enhanced ultrasonography maximum intensity projection imaging. JACC Cardiovasc. Imaging.

[128] Moguillansky D., Leng X., Carson A., Lavery L., Schwartz A., Chen X., Villanueva F.S. (2011). Quantification of plaque neovascularization using contrast ultrasound: A histologic validation. Eur. Heart J..

[129] Tian J., Hu S., Sun Y., Yu H., Han X., Cheng W., Ban X., Zhang S., Yu B., Jang I.K. (2013). Vasa vasorum and plaque progression, and responses to atorvastatin in a rabbit model of atherosclerosis: Contrast-enhanced ultrasound imaging and intravascular ultrasound study. Heart.

[130] Deyama J., Nakamura T., Takishima I., Fujioka D., Kawabata K., Obata J.E., Watanabe K., Watanabe Y., Saito Y., Mishina H. (2013). Contrast-enhanced ultrasound imaging of carotid plaque neovascularization is useful for identifying high-risk patients with coronary artery disease. Circ. J..

[131] Vavuranakis M., Sigala F., Vrachatis D.A., Papaioannou T.G., Filis K., Kavantzas N., Kalogeras K.I., Massoura C., Toufektzian L., Kariori M.G. (2013). Quantitative analysis of carotid plaque vasa vasorum by CEUS and correlation with histology after endarterectomy. VASA Z. Gefasskrankh..

[132] Kubo T., Tanaka A., Ino Y., Kitabata H., Shiono Y., Akasaka T. (2014). Assessment of coronary atherosclerosis using optical coherence tomography. J. Atheroscler. Thromb..

[133] Kume T., Akasaka T., Kawamoto T., Watanabe N., Toyota E., Neishi Y., Sukmawan R., Sadahira Y., Yoshida K. (2005). Assessment of coronary intima—Media thickness by optical coherence tomography: Comparison with intravascular ultrasound. Circ. J..

[134] Kitabata H., Tanaka A., Kubo T., Takarada S., Kashiwagi M., Tsujioka H., Ikejima H., Kuroi A., Kataiwa H., Ishibashi K. (2010). Relation of microchannel structure identified by optical coherence tomography to plaque vulnerability in patients with coronary artery disease. Am. J. Cardiol..

[135] Cheng K.H., Sun C., Vuong B., Lee K.K., Mariampillai A., Marotta T.R., Spears J., Montanera W.J., Herman P.R., Kiehl T.R. (2012). Endovascular optical coherence tomography intensity kurtosis: Visualization of vasa vasorum in porcine carotid artery. Biomed. Opt. Express.

[136] Tearney G.J., Regar E., Akasaka T., Adriaenssens T., Barlis P., Bezerra H.G., Bouma B., Bruining N., Cho J.M., Chowdhary S. (2012). Consensus standards for acquisition, measurement, and reporting of intravascular optical coherence tomography studies: A report from the International Working Group for Intravascular Optical Coherence Tomography Standardization and Validation. J. Am. Coll. Cardiol..

[137] Nishimiya K., Matsumoto Y., Takahashi J., Uzuka H., Odaka Y., Nihei T., Hao K., Tsuburaya R., Ito K., Shimokawa H. (2014). *In vivo* visualization of adventitial vasa vasorum of the human coronary artery on optical frequency domain imaging. Validation study. Circ. J..

[138] Aoki T., Rodriguez-Porcel M., Matsuo Y., Cassar A., Kwon T.G., Franchi F., Gulati R., Kushwaha S.S., Lennon R.J., Lerman L.O. (2015). Evaluation of coronary adventitial vasa vasorum using 3D optical coherence tomography—Animal and human studies. Atherosclerosis.

[139] Dobrucki L.W., Sinusas A.J. (2010). PET and SPECT in cardiovascular molecular imaging. Nat. Rev. Cardiol..

[140] Tawakol A., Migrino R.Q., Bashian G.G., Bedri S., Vermylen D., Cury R.C., Yates D., LaMuraglia G.M., Furie K., Houser S. (2006). *In vivo*
^18^F-fluorodeoxyglucose positron emission tomography imaging provides a noninvasive measure of carotid plaque inflammation in patients. J. Am. Coll. Cardiol..

[141] Folco E.J., Sheikine Y., Rocha V.Z., Christen T., Shvartz E., Sukhova G.K., di Carli M.F., Libby P. (2011). Hypoxia but not inflammation augments glucose uptake in human macrophages: Implications for imaging atherosclerosis with ^18^Fluorine-labeled 2-deoxy-d-glucose positron emission tomography. J. Am. Coll. Cardiol..

[142] Rodriguez-Porcel M., Cai W., Gheysens O., Willmann J.K., Chen K., Wang H., Chen I.Y., He L., Wu J.C., Li Z.B. (2008). Imaging of VEGF receptor in a rat myocardial infarction model using PET. J. Nucl. Med..

[143] Meoli D.F., Sadeghi M.M., Krassilnikova S., Bourke B.N., Giordano F.J., Dione D.P., Su H., Edwards D.S., Liu S., Harris T.D. (2004). Noninvasive imaging of myocardial angiogenesis following experimental myocardial infarction. J. Clin. Investig..

[144] Janssen M.L., Oyen W.J., Dijkgraaf I., Massuger L.F., Frielink C., Edwards D.S., Rajopadhye M., Boonstra H., Corstens F.H., Boerman O.C. (2002). Tumor targeting with radiolabeled α_v_β_3_ integrin binding peptides in a nude mouse model. Cancer Res..

[145] Kopka K., Breyholz H.J., Wagner S., Law M.P., Riemann B., Schroer S., Trub M., Guilbert B., Levkau B., Schober O. (2004). Synthesis and preliminary biological evaluation of new radioiodinated MMP inhibitors for imaging MMP activity *in vivo*. Nucl. Med. Biol..

[146] Furumoto S., Takashima K., Kubota K., Ido T., Iwata R., Fukuda H. (2003). Tumor detection using ^18^F-labeled matrix metalloproteinase-2 inhibitor. Nucl.Med. Biol..

[147] Schafers M., Riemann B., Kopka K., Breyholz H.J., Wagner S., Schafers K.P., Law M.P., Schober O., Levkau B. (2004). Scintigraphic imaging of matrix metalloproteinase activity in the arterial wall *in vivo*. Circulation.

[148] Matter C.M., Schuler P.K., Alessi P., Meier P., Ricci R., Zhang D., Halin C., Castellani P., Zardi L., Hofer C.K. (2004). Molecular imaging of atherosclerotic plaques using a human antibody against the extra-domain B of fibronectin. Circ. Res..

[149] Kuge Y., Takai N., Ogawa Y., Temma T., Zhao Y., Nishigori K., Ishino S., Kamihashi J., Kiyono Y., Shiomi M. (2010). Imaging with radiolabelled anti-membrane type 1 matrix metalloproteinase (MT1-MMP) antibody: Potentials for characterizing atherosclerotic plaques. Eur. J. Nucl. Med. Mol. Imaging.

[150] Aoki S., Aoki K., Ohsawa S., Nakajima H., Kumagai H., Araki T. (1999). Dynamic MR imaging of the carotid wall. J. Magn. Reson. Imaging JMRI.

[151] Winter P.M., Morawski A.M., Caruthers S.D., Fuhrhop R.W., Zhang H., Williams T.A., Allen J.S., Lacy E.K., Robertson J.D., Lanza G.M. (2003). Molecular imaging of angiogenesis in early-stage atherosclerosis with α_v_β_3_-integrin-targeted nanoparticles. Circulation.

[152] Cai K., Caruthers S.D., Huang W., Williams T.A., Zhang H., Wickline S.A., Lanza G.M., Winter P.M. (2010). MR molecular imaging of aortic angiogenesis. JACC Cardiovasc. Imaging.

[153] Kerwin W., Hooker A., Spilker M., Vicini P., Ferguson M., Hatsukami T., Yuan C. (2003). Quantitative magnetic resonance imaging analysis of neovasculature volume in carotid atherosclerotic plaque. Circulation.

[154] Kerwin W.S., Oikawa M., Yuan C., Jarvik G.P., Hatsukami T.S. (2008). MR imaging of adventitial vasa vasorum in carotid atherosclerosis. Magn. Reson. Med..

[155] Sun J., Song Y., Chen H., Kerwin W.S., Hippe D.S., Dong L., Chen M., Zhou C., Hatsukami T.S., Yuan C. (2013). Adventitial perfusion and intraplaque hemorrhage: A dynamic contrast-enhanced MRI study in the carotid artery. Stroke J. Cereb. Circ..

[156] Grobner T. (2006). Gadolinium—A specific trigger for the development of nephrogenic fibrosing dermopathy and nephrogenic systemic fibrosis?. Nephrol. Dial. Transpl..

[157] Feinstein S.B. (2006). Contrast ultrasound imaging of the carotid artery vasa vasorum and atherosclerotic plaque neovascularization. J. Am. Coll. Cardiol..

[158] Decano J.L., Moran A.M., Ruiz-Opazo N., Herrera V.L. (2011). Molecular imaging of vasa vasorum neovascularization via DEspR-targeted contrast-enhanced ultrasound micro-imaging in transgenic atherosclerosis rat model. Mol. Imaging Biol. MIB.

[159] Baron Toaldo M., Salvatore V., Marinelli S., Palama C., Milazzo M., Croci L., Venerandi L., Cipone M., Bolondi L., Piscaglia F. (2015). Use of VEGFR-2 targeted ultrasound contrast agent for the early evaluation of response to sorafenib in a mouse model of hepatocellular carcinoma. Mol. Imaging Biol. MIB.

[160] Araldi E., Chamorro-Jorganes A., van Solingen C., Fernandez-Hernando C., Suarez Y. (2013). Therapeutic potential of modulating microRNAs in atherosclerotic vascular disease. Curr. Vasc. Pharmacol..

[161] O’Reilly M.S., Holmgren L., Shing Y., Chen C., Rosenthal R.A., Moses M., Lane W.S., Cao Y., Sage E.H., Folkman J. (1994). Angiostatin: A novel angiogenesis inhibitor that mediates the suppression of metastases by a Lewis lung carcinoma. Cell.

[162] O’Reilly M.S., Boehm T., Shing Y., Fukai N., Vasios G., Lane W.S., Flynn E., Birkhead J.R., Olsen B.R., Folkman J. (1997). Endostatin: An endogenous inhibitor of angiogenesis and tumor growth. Cell.

[163] Moulton K.S., Heller E., Konerding M.A., Flynn E., Palinski W., Folkman J. (1999). Angiogenesis inhibitors endostatin or TNP-470 reduce intimal neovascularization and plaque growth in apolipoprotein E-deficient mice. Circulation.

[164] Moulton K.S., Vakili K., Zurakowski D., Soliman M., Butterfield C., Sylvin E., Lo K.M., Gillies S., Javaherian K., Folkman J. (2003). Inhibition of plaque neovascularization reduces macrophage accumulation and progression of advanced atherosclerosis. Proc. Natl. Acad. Sci. USA.

[165] Eriksson K., Magnusson P., Dixelius J., Claesson-Welsh L., Cross M.J. (2003). Angiostatin and endostatin inhibit endothelial cell migration in response to FGF and VEGF without interfering with specific intracellular signal transduction pathways. FEBS Lett..

[166] Stefanadis C., Toutouzas K., Stefanadi E., Kolodgie F., Virmani R., Kipshidze N. (2006). First experimental application of bevacizumab-eluting PC coated stent for inhibition of vasa vasorum of atherosclerotic plaque: Angiographic results in a rabbit atheromatic model. Hell. J. Cardiol. HJC.

[167] Luttun A., Tjwa M., Moons L., Wu Y., Angelillo-Scherrer A., Liao F., Nagy J.A., Hooper A., Priller J., de Klerck B. (2002). Revascularization of ischemic tissues by PlGF treatment, and inhibition of tumor angiogenesis, arthritis and atherosclerosis by anti-Flt1. Nat. Med..

[168] Khurana R., Moons L., Shafi S., Luttun A., Collen D., Martin J.F., Carmeliet P., Zachary I.C. (2005). Placental growth factor promotes atherosclerotic intimal thickening and macrophage accumulation. Circulation.

[169] Fischer C., Jonckx B., Mazzone M., Zacchigna S., Loges S., Pattarini L., Chorianopoulos E., Liesenborghs L., Koch M., de Mol M. (2007). Anti-PlGF inhibits growth of VEGF(R)-inhibitor-resistant tumors without affecting healthy vessels. Cell.

[170] Johnson M.D., Kim H.R., Chesler L., Tsao-Wu G., Bouck N., Polverini P.J. (1994). Inhibition of angiogenesis by tissue inhibitor of metalloproteinase. J. Cell. Physiol..

[171] Gossl M., Herrmann J., Tang H., Versari D., Galili O., Mannheim D., Rajkumar S.V., Lerman L.O., Lerman A. (2009). Prevention of vasa vasorum neovascularization attenuates early neointima formation in experimental hypercholesterolemia. Basic Res. Cardiol..

[172] Kampschulte M., Gunkel I., Stieger P., Sedding D.G., Brinkmann A., Ritman E.L., Krombach G.A., Langheinrich A.C. (2014). Thalidomide influences atherogenesis in aortas of ApoE^−/−^/LDLR^−/−^ double knockout mice: A nano-CT study. Int. J. Cardiovasc. Imaging.

[173] Dashwood M.R., Barker S.G., Muddle J.R., Yacoub M.H., Martin J.F. (1993). [125I]-endothelin-1 binding to vasa vasorum and regions of neovascularization in human and porcine blood vessels: A possible role for endothelin in intimal hyperplasia and atherosclerosis. J. Cardiovasc. Pharmacol..

[174] Herrmann J., Best P.J., Ritman E.L., Holmes D.R., Lerman L.O., Lerman A. (2002). Chronic endothelin receptor antagonism prevents coronary vasa vasorum neovascularization in experimental hypercholesterolemia. J. Am. Coll. Cardiol..

[175] Winter P.M., Neubauer A.M., Caruthers S.D., Harris T.D., Robertson J.D., Williams T.A., Schmieder A.H., Hu G., Allen J.S., Lacy E.K. (2006). Endothelial α_v_β_3_ integrin-targeted fumagillin nanoparticles inhibit angiogenesis in atherosclerosis. Arterioscler. Thromb. Vasc. Biol..

[176] Drinane M., Mollmark J., Zagorchev L., Moodie K., Sun B., Hall A., Shipman S., Morganelli P., Simons M., Mulligan-Kehoe M.J. (2009). The antiangiogenic activity of rPAI-1_23_ inhibits vasa vasorum and growth of atherosclerotic plaque. Circ. Res..

[177] Mollmark J., Ravi S., Sun B., Shipman S., Buitendijk M., Simons M., Mulligan-Kehoe M.J. (2011). Antiangiogenic activity of rPAI-1_23_ promotes vasa vasorum regression in hypercholesterolemic mice through a plasmin-dependent mechanism. Circ. Res..

[178] Langheinrich A.C., Sedding D.G., Kampschulte M., Moritz R., Wilhelm J., Haberbosch W.G., Ritman E.L., Bohle R.M. (2009). 3-Deazaadenosine inhibits vasa vasorum neovascularization in aortas of ApoE^−/−^/LDLR^−/−^ double knockout mice. Atherosclerosis.

[179] Tsai H.T., Marshall J.L., Weiss S.R., Huang C.Y., Warren J.L., Freedman A.N., Fu A.Z., Sansbury L.B., Potosky A.L. (2013). Bevacizumab use and risk of cardiovascular adverse events among elderly patients with colorectal cancer receiving chemotherapy: A population-based study. Ann. Oncol..

[180] Colon G.P., Deveikis J.P., Dickinson L.D. (1999). Revascularization of occluded internal carotid arteries by hypertrophied vasa vasorum: Report of four cases. Neurosurgery.

[181] Shanahan C.M., Weissberg P.L. (1999). Smooth muscle cell phenotypes in atherosclerotic lesions. Curr. Opin. Lipidol..

[182] Wilson S.H., Herrmann J., Lerman L.O., Holmes D.R., Napoli C., Ritman E.L., Lerman A. (2002). Simvastatin preserves the structure of coronary adventitial vasa vasorum in experimental hypercholesterolemia independent of lipid lowering. Circulation.

[183] Urbich C., Dernbach E., Zeiher A.M., Dimmeler S. (2002). Double-edged role of statins in angiogenesis signaling. Circ. Res..

[184] Chaldakov G.N., Stankulov I.S., Fiore M., Ghenev P.I., Aloe L. (2001). Nerve growth factor levels and mast cell distribution in human coronary atherosclerosis. Atherosclerosis.

[185] Asanome A., Kawabe J., Matsuki M., Kabara M., Hira Y., Bochimoto H., Yamauchi A., Aonuma T., Takehara N., Watanabe T. (2014). Nerve growth factor stimulates regeneration of perivascular nerve, and induces the maturation of microvessels around the injured artery. Biochem. Biophys. Res. Commun..

[186] Mollmark J.I., Park A.J., Kim J., Wang T.Z., Katzenell S., Shipman S.L., Zagorchev L.G., Simons M., Mulligan-Kehoe M.J. (2012). Fibroblast growth factor-2 is required for vasa vasorum plexus stability in hypercholesterolemic mice. Arterioscler. Thromb. Vasc. Biol..

[187] Lindner V., Lappi D.A., Baird A., Majack R.A., Reidy M.A. (1991). Role of basic fibroblast growth factor in vascular lesion formation. Circ. Res..

[188] Six I., Mouquet F., Corseaux D., Bordet R., Letourneau T., Vallet B., Dosquet C.C., Dupuis B., Jude B., Bertrand M.E. (2004). Protective effects of basic fibroblast growth factor in early atherosclerosis. Growth Factors.

[189] Che J., Okigaki M., Takahashi T., Katsume A., Adachi Y., Yamaguchi S., Matsunaga S., Takeda M., Matsui A., Kishita E. (2011). Endothelial FGF receptor signaling accelerates atherosclerosis. Am. J. Physiol. Heart Circ. Physiol..

[190] Shen W., Shi H.M., Fan W.H., Luo X.P., Jin B., Li Y. (2011). The effects of simvastatin on angiogenesis: Studied by an original model of atherosclerosis and acute myocardial infarction in rabbit. Mol. Biol. Rep..

